# Benefits of Pyrenemethanol as an Initiator for the ROP of Morpholine-2,5-Diones: Fluorescence Probing of Nanoparticle Degradation and Cellular Uptake

**DOI:** 10.1021/acspolymersau.6c00097

**Published:** 2026-07-08

**Authors:** Johanna Schreiber, Lea C. Klepsch, Jana Ismail, David Pretzel, Antje Vollrath, Christine Weber, Ulrich S. Schubert

**Affiliations:** † Laboratory of Organic and Macromolecular Chemistry (IOMC), 9378 Friedrich Schiller University Jena, Humboldtstr. 10, Jena 07743, Germany; ‡ Jena Center for Soft Matter (JCSM), Friedrich Schiller University Jena, Philosophenweg 7, Jena 07743, Germany

**Keywords:** poly(ester amide), morpholine-2,5-dione, pyrene, ring-opening polymerization, nanoparticle, degradation, fluorescence

## Abstract

1-Pyrenemethanol was used as an initiator for the ring-opening polymerization (ROP) of three morpholine-2,5-diones, which are based on the naturally occurring amino acids l-phenylalanine, l-isoleucine, and l-valine. A catalytic system consisting of 1,8-diazabicyclo[5.4.0]­undec-7-ene (DBU) and 1-(3,5-bis­(trifluoromethyl)­phenyl)-3-cyclohexylthiourea (TU) was applied for the polymerizations at room temperature. Kinetic studies demonstrated controlled reaction conditions and the absence of transesterification under the selected parameters. By means of ^1^H NMR spectroscopy and size exclusion chromatography (SEC), monomer conversion values of about 90% were observed during the considered reaction times of up to 1 h, whereby the dispersity remained below 1.15. The covalent attachment of the pyrene α-end group to the respective poly­(morpholine-2,5-dione)­s (PEAs) was proven by ^1^H NMR spectroscopy, SEC using a diode array detector, matrix-assisted laser desorption/ionization time-of-flight mass spectrometry (MALDI-TOF MS), and fluorescence spectroscopy. The PEAs formed spherical nanoparticles with uniform size distribution via nanoprecipitation, which were subjected to degradation studies using proteinase K. Due to the characteristic change in the vibronic fine structure of the fluorescence spectrum of pyrene with the changing molecular environment, this process could be observed by fluorescence spectroscopy. The received data were validated by dynamic light scattering (DLS) measurements. Furthermore, the pyrene end group served as a fluorescent label for cellular uptake studies in L929 cells monitored by confocal laser scanning microscopy (CLSM). This report demonstrates the added value of the introduction of the fluorescence-active pyrene into the polymer structure, enabling access to additional information about the polymer and its nanoparticles without additional synthesis effort.

## Graphical abstract

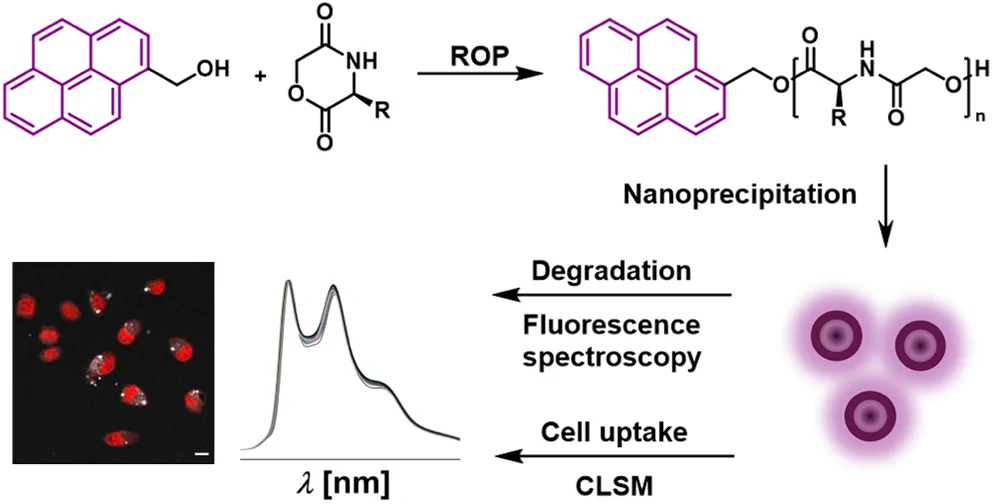

## 1 Introduction

Biocompatible poly­(ester amide)­s are the focus of current research as an alternative to polyesters, which are frequently used in biomedical applications, for example, as materials for nanoparticulate drug delivery systems.^1–6^ In addition to ester groups that are cleavable under acidic, basic, or enzymatic conditions,^7,8^ such polymers also possess more stable amide bonds in the backbone. This circumstance allows for the adjustment of the degradability and drug release of nanoparticulate systems regarding their intended use.^2,9,10^ These processes may also be influenced by changing the polymer composition and, therefore, the ratio of ester-to-amide bonds in the backbone.^4,5,7^ The introduction of different side chains is another option to adjust the degradability or interaction with the drug.^7,8^

Poly­(morpholine-2,5-dione)­s (PEAs) represent a subclass of poly­(ester amide)­s that can be obtained by ring-opening polymerization (ROP) of the corresponding cyclic monomers, i.e., morpholine-2,5-diones.^3^ Organic or organometallic substances, as well as enzymes, are used for the catalysis of these reactions.^11,12^ While polymer syntheses using enzymes or metal catalysts require very high temperatures,^13–18^ organocatalytic approaches can be carried out at room temperature, which also enables the use of temperature-sensitive materials.^19^ Especially since the introduction of bicatalytic systems based on 1,5,7-triazabicyclo[4.4.0]­dec-5-ene (TBD) or 1,8-diazabicyclo[5.4.0]­undec-7-ene (DBU) and thiourea derivatives, it has been possible to achieve monomer conversion values above 90% while maintaining narrow molar mass distributions.^11,19–22^ Thus, well-defined polymers can be obtained under comparatively mild reaction conditions. The absence of a toxic metal catalyst such as tin­(II) 2-ethylhexanoate represents a further advantage regarding the application of PEAs in the biomedical field as nanoparticulate drug delivery systems.^12^

The end group of the PEA is determined by the initiator used for the ROP, that is, a primary alcohol. Mostly, benzyl alcohol has been utilized for this purpose.^2,10,20,21,23–25^ Polymers with hydroxyl end groups as macroinitiators have enabled access to block copolymers comprising PEAs.^11,26^ Also, diols have been applied as bifunctional ROP initiators to yield homotelechelic PEAs.^6^ We recently reported the use of a cyclooctyne-functionalized alcohol for the initiation of the ROP to enable access to sophisticated block copolymer architectures with targeting ligands.^19^

Many recent studies of PEAs emphasize the use of these materials in various biomedical contexts. The question arises whether the used ROP initiator could directly shed light on relevant material properties, such as the degradability of the PEA and its effect on cells. Both are usually investigated at the beginning of research directed toward different biomedical applications, including tissue engineering or nanoparticulate polymeric carriers.

Fluorescence-based analysis methods are often necessary for this purpose.^27,28^ For instance, confocal laser scanning microscopy (CLSM) relies on specialized fluorescent dyes such as fluorescein, Cy3, Cy5, rhodamine derivatives, or Alexa Fluor dyes to investigate, e.g., the cellular uptake of polymers or nanocarriers.^29^ These dyes are favored for their high quantum yields, photostability, and compatibility with standard lasers. However, they are often expensive and require postpolymerization modifications, followed by extensive purification steps to remove the uncoupled dye, which increases the synthetic effort and consumes time. The encapsulation of a fluorescent dye into the nanoparticle (NP)^30^ represents an alternative that tracks the location of an encapsulated guest molecule, which is not necessarily colocated with the polymeric carrier due to its release by leakage or degradation.^31^ This can be avoided when a fluorescent label is directly introduced during polymer synthesis through the selection of a suitable initiator. Pyrene-functionalized alcohols have already been applied to initiate the ROP of lactones^32,33^ and oxazepanones.^34^ In fact, the first report of the organo-catalyzed ROP of morpholine-2,5-diones in 2005 by Pratt et al.^35,36^ relied on the use of pyrenebutanol as an initiator. Pyrene is well-known in polymer science as a fluorescent probe for the determination of the critical micelle concentration (CMC).^37,38^ Its use is based on the change of the vibronic band intensities in the fluorescence spectrum within the molecular environment, as first investigated by Nakajima in the 1970s.^39–41^ Since the description of pyrene as a fluorescent dye for the CMC determination by Kalyanasundaram and Thomas in 1977,^37^ the so-called 3:1 (or 1:3) method has become established and is still used today.^42–46^

To the best of our knowledge, the possibility of using (covalently bound) pyrene to observe the opposite process, i.e., NP degradation, has never been investigated before. For the development of such a method, three pyrene end-functionalized PEAs (Py-PEAs) were synthesized by organo-catalyzed ROP (Scheme 1). Thus, the monomers PheG, ValG, and IleG were used, which are based on the naturally occurring α-amino acids l-phenylalanine, l-valine, and l-isoleucine. 1-Pyrenemethanol (PyOH) was applied as an initiator for the DBU/1-(3,5-bis­(trifluoromethyl)­phenyl)-3-cyclohexylthiourea (TU)-catalyzed polymerization, yielding **Py-PPheG**, **Py-PIleG**, and **Py-PValG**. Subsequent to end-group confirmation, NPs were formulated by nanoprecipitation. Proteinase K-mediated degradation studies were monitored by fluorescence spectroscopy. The obtained results were compared to observations made by dynamic light scattering (DLS), which represents the state-of-the-art technique for that purpose. The internalization of the NPs into L929 cells was also investigated using CLSM to demonstrate an added benefit of pyrene as an α-end group.

**Scheme 1:**
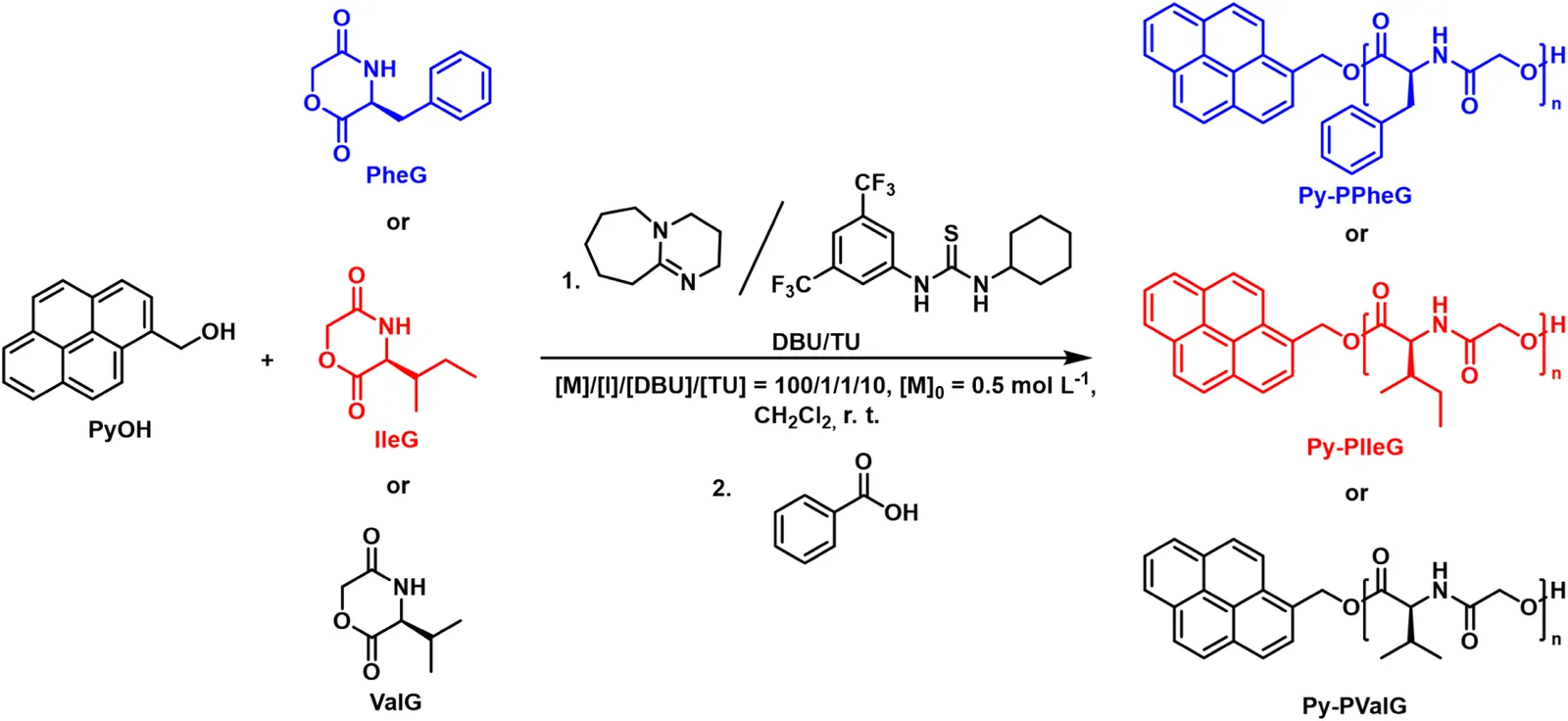
Schematic Representation of the Synthesis Approach toward the Polymers **Py-PPheG**, **Py-PIleG**, and **Py-PValG** by using the Monomers PheG, IleG, and ValG

## 2 Experimental Section

### 2.1 Polymer Synthesis and Characterization

#### 2.1.1 Materials

l-Phenylalanine, l-isoleucine, as well as l-valine (>98%) were purchased from TCI. Chloroacetyl chloride (≥99.0%), triethylamine (NEt_3_, ≥99.5%), 1,8-diazabicyclo[5.4.0]­undec-7-ene (DBU, ≥99.0%), benzoic acid (≥99.5%), and PyOH (98%) were supplied by Sigma-Aldrich. TU was synthesized following a procedure by Pratt et al.^33^ The solvents for the synthesis were dried using a solvent purification system (MB SPS-800, MBRAUN). All other chemicals were purchased from the usual suppliers and used without further purification.

#### 2.1.2 Instrumentation

Polymerizations were performed under a nitrogen atmosphere in a UNIlab Plus glovebox from MBRAUN. The device was equipped with a vacuum pump, a UNIlab SP gas purification system, and MB B-L-03 HEPA filters.

Proton nuclear magnetic resonance (^1^H NMR) spectroscopy was measured using a 300 MHz Avance I spectrometer (Bruker), equipped with a dual ^1^H and ^13^C sample head and a BACS 120 autosampler. The spectra were measured in deuterated dimethyl sulfoxide (DMSO-*d*_6_), CD_2_Cl_2_, or CDCl_3_. Chemical shifts (δ) are given in ppm with reference to the residual solvent signals.

Size exclusion chromatography (SEC) measurements were performed using an Agilent 1200 series system, which was equipped with a G1329A autosampler, a PSS degasser, a G1310A pump, as well as a Techlab oven (40 °C). A PSS Gram guard 30 Å and a PSS Gram guard 1000 Å column were placed in series (10 μm particle size). Signals were detected by a G7162A refractive index detector (RID) and a G1315D diode array detector (DAD). A solution of 0.21% (w/w) LiCl in *N,N*-dimethylacetamide (DMAc) was used as an eluent. The adjusted flow rate was 1 mL min^–1^. Polystyrene (PS, Agilent Technologies, 400 to 1,000,000 g mol^–1^) was used as the calibration standard for the determination of the molar masses.

Matrix-assisted laser desorption/ionization time-of-flight mass spectrometry (MALDI-TOF MS) was carried out using a RapifleX MALDI-TOF/TOF system (Bruker Daltonics), which was equipped with a ScoutMTP II ion source and a Smartbeam 3D laser (355 nm wavelength). Spectra were recorded in positive reflector mode. *trans*-2-[3-(4-*tert*-Butylphenyl)-2-methyl-2-propenylidene]­malononitrile (DCTB) or dithranol was applied as the matrix, and sodium trifluoroacetate (NaTFA) as the doping salt. The received data were processed with the manufacturer’s software FlexAnalysis 4.0. FleXstandard (Bruker) was used as the calibration standard.

#### 2.1.3 Monomer Syntheses

The monomers PheG, IleG, and ValG were synthesized as previously described.^11,19^

In brief, the corresponding l-amino acid was suspended in dry tetrahydrofuran (THF) under an argon atmosphere. Subsequently, 1 equiv of NEt_3_ was added to the suspension, which was stirred at room temperature for 20 min. The reaction mixture was cooled to −5 °C. 1.2 equiv of chloroacetyl chloride in dry THF was added dropwise over a period of 20 min, while the temperature was kept below 0 °C. After stirring for 30 min, the suspension was allowed to warm up to room temperature and stirred for another 1.5 h, diluted with distilled water, and washed with brine. The combined aqueous phases were extracted with ethyl acetate. The organic phases were dried over Na_2_SO_4_, filtered, concentrated under reduced pressure, and suspended in diethyl ether. After filtration, the solvent was removed under reduced pressure. The crude product was used without further purification for the following cyclization.

For that purpose, 8 equiv of NaHCO_3_ was suspended in *N,N*-dimethylformamide (DMF) and heated to 60 °C. The crude product dissolved in DMF was added dropwise over a period of 30 min, and the reaction mixture was stirred overnight at 60 °C. After filtration, the solution was concentrated under reduced pressure. The residue was suspended in CHCl_3_ and filtered. The filtrate was dried over MgSO_4_. The solvent was removed under reduced pressure. The product was recrystallized using a 1:1 (v/v) mixture of *n*-hexane and ethyl acetate and dried *in vacuo*.

#### 2.1.4 Ring-Opening Polymerization

All polymers were synthesized in accordance with previously published procedures.^11,19^ Preheated vials were cooled to room temperature under a continuous argon flow and used as reaction vessels. The ratio of monomer to initiator to DBU to TU ([M]/[I]/[DBU]/[TU]) was 100/1/1/10, and the monomer concentration was 0.5 mol L^–1^.

##### 2.1.4.1 General Procedure for the Performance of the Kinetic Studies

The monomer and TU were dissolved in dry CH_2_Cl_2_. Subsequently, the required volume of a stock solution of DBU and PyOH was added to initiate the polymerization. The reaction mixture was stirred at room temperature, and samples were taken at specific time intervals. Those aliquots were each quenched with at least 10 equiv of benzoic acid and analyzed by ^1^H NMR spectroscopy and SEC.

Kinetic Study Using PheG:

100.3 mg of PheG (488.8 μmol), 1.135 mg of PyOH (4.9 μmol), 0.73 μL of DBU (0.74 mg; 4.9 μmol), 18.0 mg of TU (48.6 μmol), and a total volume of 975 μL dry CH_2_Cl_2_ were used.

Kinetic Study Using IleG:

150.0 mg of IleG (881.3 μmol), 2.045 of mg PyOH (8.8 μmol), 1.31 μL of DBU (1.28 mg; 8.4 μmol),32.6 mg of TU (88.0 μmol), and a total volume of 1.762 mL dry CH_2_Cl_2_ were used.

Kinetic Study Using ValG:

150.5 mg of ValG (957.6 μmol), 2.22 mg of PyOH (9.5 μmol), 1.42 μL of DBU (1.45 mg; 9.5 μmol), 35.4 mg of TU (95.6 μmol), and a total volume of 1.910 mL dry CH_2_Cl_2_ were used.

##### 2.1.4.2 General Polymer Synthesis Procedure

Monomer and TU were dissolved in a specific volume of dry CH_2_Cl_2_, taking into account the required amount of DBU/PyOH stock solution. The corresponding volume of the latter was added subsequently. The reaction was stirred at room temperature. The reaction times and, thus, the conversion were selected individually based on the results of the kinetic studies. 10 equiv of benzoic acid was added to terminate the polymerization. An aliquot was taken and analyzed by ^1^H NMR spectroscopy to determine the monomer conversion and thus the degree of polymerization (DP_theo_). The resulting polymer was purified by precipitating several times in cold diethyl ether (−80 °C) and dried *in vacuo*.

Synthesis of **Py-PPheG**:

1.316 g of PheG (6.413 mmol), 14.90 mg of PyOH (64.1 μmol), 9.57 μL of DBU (9.76 mg; 64.1 μmol), and 237.5 mg of TU (641.3 μmol) were used in an overall volume of 12.82 mL of dry CH_2_Cl_2_. 86 mg of benzoic acid (704.2 μmol) was used at a reaction time of 45 min to terminate the polymerization.

Yield: 950 mg of a white solid. Conversion: 87%. DP_theo_: 87.

^1^H NMR (300 MHz, CD_2_Cl_2_): δ = 2.86–3.30 (m, 2 H, CH_2_, benzyl substituent); 3.91–4.86 (m, 3 H, CH, CH_2_, backbone); 5.84 (m, CH_2_, pyrene end group); 6.95–7.35 (m, 5 H, benzyl substituent); 7.59–7.90 (m, 1 H, NH); 7.97–8.30 (m, CH, pyrene end group) ppm.

SEC (DMAc, 0.21% (w/w) LiCl; RID; PS calibration): *M*_n_ = 19,900 g mol^–1^. *Đ* = 1.15.

MALDI-TOF MS (DCTB, NaTFA): [C_17_H_11_O­(C_11_H_11_NO_3_)_*n*_H + Na]^+^, [HO­(C_11_H_11_NO_3_)_*n*_H + Na]^+^, [NaO­(C_11_H_11_NO_3_)_*n*_H + Na]^+^, and [(C_11_H_11_NO_3_)_*n*_ + Na]^+^ observed.

Synthesis of **Py-PIleG**:

1000 mg of IleG (5.875 mmol), 13.63 mg of PyOH (58.7 μmol), 8.73 μL of DBU (8.91 mg; 58.5 μmol), and 217.6 mg of TU (587.5 μmol) were used in an overall volume of 11.755 mL of dry CH_2_Cl_2_. 72 mg of benzoic acid (589.6 μmol) was used at a reaction time of 60 min to terminate the reaction.

Yield: 800 mg of a white solid. Conversion: 92%. DP_theo_: 92.

^1^H NMR (300 MHz, CD_2_Cl_2_): δ = 0.64–1.08 (m, 6 H, CH_3_, *sec*-butyl substituent); 1.10–1.80 (m, 3 H, CH_2_, *sec*-butyl substituent, water); 1.82–2.17 (m, 1 H, CH, *sec*-butyl substituent); 4.03–4.94 (m, 3H, CH, CH_2_, backbone); 5.78–6.01 (m, CH_2_, pyrene end group); 7.42–7.78 (m, 1 H, NH); 8.00–8.38 (m, CH, pyrene end group) ppm.

SEC (DMAc, 0.21% (w/w) LiCl; RID; PS calibration): *M*_n_ = 25,400 g mol^–1^. *Đ* = 1.12.

MALDI-TOF MS (dithranol, NaTFA): [C_17_H_11_O­(C_8_H_13_NO_3_)_*n*_H + Na]^+^, [HO­(C_8_H_13_NO_3_)_*n*_H + Na]^+^; and [(C_8_H_13_NO_3_)_*n*_ + Na]^+^ observed.

Synthesis of **Py-PValG**:

690 mg of ValG (4.390 mmol), 10.2 mg of PyOH (43.9 μmol), 6.55 μL of DBU (6.68 mg; 43.9 μmol), and 162.6 mg of TU (493.0 μmol) were used in an overall volume of 8.78 mL of dry CH_2_Cl_2_. 54 mg of benzoic acid (442.2 μmol) was used at a reaction time of 60 min to terminate the reaction.

Yield: 427 mg of a white solid. Conversion: 83%. DP_theo_: 83.

^1^H NMR (300 MHz, CD_2_Cl_2_): δ = 0.74–1.10 (m, 6 H, CH_3_, *iso*-propyl substituent); 2.08–2.34 (m, 1 H, CH, *iso*-propyl substituent); 4.05–4.33 (m, 1 H, CH, backbone); 4.35–4.91 (m, 2 H, CH_2_, backbone); 5.81–5.99 (m, CH_2_, pyrene end group); 7.42–7.70 (m, 1H, NH); 8.01–8.34 (m, CH, pyrene end group) ppm.

SEC (DMAc, 0.21% (w/w) LiCl; RID; PS calibration): *M*_n_ = 19,200 g mol^–1^. *Đ* = 1.14.

MALDI-TOF MS (dithranol, NaTFA): [C_17_H_11_O­(C_7_H_11_NO_3_)_*n*_H + Na]^+^, [HO­(C_7_H_11_NO_3_)_*n*_H + Na]^+^; and [(C_7_H_11_NO_3_)_*n*_ + Na]^+^ observed.

### 2.2 Nanoparticle Formulation and Characterization

#### 2.2.1 Materials

Purified water was obtained from a GenPure ultrapure water purification system (Thermo Scientific) and was used in all stages of NP preparation, purification, and characterization studies. Poly­(vinyl alcohol) (PVA, Mowiol 4–88, *M*_w_ = 31,000 g mol^–1^) and proteinase K from *Tritirachium* were obtained from Sigma-Aldrich. THF (99.8+%, HPLC grade) was purchased from AnalytiChem. Acetonitrile (≥99.5%) was received from Carl Roth.

#### 2.2.2 Nanoprecipitation

Formulation of NPs without PVA was performed as follows: **Py-PPheG**, **Py-PIleG**, or **Py-PValG** (around 1 mg) was dissolved in THF at a concentration of 2 mg mL^–1^. Separately, 3 mL of purified water was placed into a glass vial. The polymer solution was transferred into a 1 mL syringe fitted with a 21Gx43/4 (0.8 × 120 mm) cannula and mounted on a syringe pump (Aladdin AL1000-220, World Precision Instruments). The cannula was bent to a 90° angle and positioned in the glass vial touching the glass wall. The polymer solution (0.5 mL) was infused into the aqueous phase at a flow rate of 2 mL min^–1^, while the solution was stirred at 800 rpm. The resulting dispersion was stirred at 800 rpm in the fume hood overnight. On the following day, the resulting particle dispersions were stored at 4 °C.

Formulation of NPs with PVA was carried out in a similar fashion using solutions of around 10 mg of polymer in acetonitrile (*c* = 10 mg mL^–1^). Separately, 8 mL of 0.3% (w/v) aqueous PVA solution was placed into a glass vial. The polymer solutions (1 mL) were transferred into a 2 mL syringe for precipitation. Subsequent to nanoprecipitation as described above, NPs were purified the following day by centrifugation at 11,000 rpm for 60 min at 20 °C using a 5804 R centrifuge (Eppendorf). After centrifugation, the supernatant was removed, and the NP pellets were redispersed in 2 mL of purified water. The suspensions were vortexed and stored at 4 °C overnight to allow equilibration. For subsequent analyses, aliquots of the dispersions were freeze-dried using a Christ Alpha 2–4 LD plus lyophilizer. The NP mass was determined with an XPR10 microbalance (Mettler Toledo), and the NP yield was calculated by dividing the actual obtained particle mass by the theoretically possible particle mass multiplied by 100. The details are summarized in Table S1.

#### 2.2.3 Nanoparticle Characterization

DLS and electrophoretic light scattering (ELS) were employed to characterize the NP populations in terms of size distribution and zeta potential (ζ) using a Zetasizer Ultra (Malvern Panalytical GmbH) equipped with a 633 nm laser. Measurements were carried out at 25 °C in polystyrene microcuvettes (Brand GmbH + Co KG), with intensity fluctuations monitored at a backscatter angle of 174.7°. The z-average hydrodynamic diameter (*d*_h_) and the polydispersity index (PDI) served as indicators of NP quality. These parameters were recorded (i) after evaporation of the organic solvent and (ii) following centrifugation and redispersion in water. Zeta potential values were measured using a DTS1070 capillary cell. For DLS and ELS measurements, NP samples were diluted 1:100 in purified water and measured five times for *d*_h_ and PDI, with 15 runs each lasting 1.68 s and three times for ζ determination. If the formulation was repeated, the data was reported as the average and standard deviation among all repeated formulations. The values are reported in Table S1.

A Sigma VP field-emission scanning electron microscope (SEM, Carl Zeiss) was employed for NP imaging. Micrographs were acquired with an InLens detector at an acceleration voltage of 4 or 6 kV. Prior to imaging, the samples were deposited onto mica substrates by drop-casting and then coated with a 4 nm layer of platinum using a CCU-010 HV sputter coater (Safematic).^30^

#### 2.2.4 Nanoparticle Degradation Studies

For the initial NP degradation tests, samples were prepared as follows: 9 μL of a **Py-PPheG**-based NP dispersion (*c* = 1.8 to 1.9 mg mL^–1^) was transferred into a cuvette, and 206 μL of proteinase K solution (*c* = 2 mg mL^–1^) or 5 μL of a 1 M NaOH solution was added. The sample was diluted with purified water to reach an overall volume of 2.465 mL. The NP concentration in the sample was 6.8 μg mL^–1^, and the proteinase K concentration was 167 μg mL^–1^ which corresponds to a particle-to-enzyme mass ratio of 1:25.^2^ For blanks, neither proteinase K nor NaOH solution was used. The samples were kept at 37 °C and analyzed at 25 °C by means of fluorescence spectroscopy and DLS at suitable time intervals.

For degradation kinetic studies, samples were prepared in a similar fashion: the NP concentration was set to 6.5 μg mL^–1^, and the enzyme concentration was set to 167 μg mL^–1^ in an overall sample volume of 2.460 mL. The particle-to-enzyme mass ratio was 1:26. The solutions were directly transferred into a fluorescence spectrometer (see below), as well as into the Malvern Zetasizer Ultra. Both instruments were operating at 37 °C. For the DLS measurements, the count rate and *d*_h_ were acquired once per time point using fixed detector settings throughout the experiment. Monitoring continued until no particles were detected by the instrument.

### 2.3 Fluorescence Spectroscopy

Fluorescence spectroscopy was performed using a quartz cuvette (1 cm pathway) in an FP-8300 device (Jasco). The measurements of the polymers in THF at a concentration of 0.01 mg mL^–1^ at 25 °C were carried out from 350 to 450 nm with a scan speed of 20 nm min^–1^ and a data interval of 0.2 nm. The extinction bandwidth was 2.5 nm, the emission bandwidth was 5 nm, and the response time was 1 s. The excitation wavelength was set to 339 nm.

Fluorescence spectroscopy of the NPs in purified water was performed in a similar fashion with small changes. For the degradation tests, measurements were performed at 25 °C, whereas the samples were stored at 37 °C between the scans. The degradation kinetic studies were carried out directly in the instrument set to 37 °C. Spectra were acquired at suitable time intervals.

### 2.4 Cellular Uptake Studies

CELLview cell culture dishes (PS, 35/10 mm, glass bottom) were procured from Greiner Bio-One. L929 cells were sourced from CLS (Cat. No. 400260). Dulbecco’s Modified Eagle Medium (DMEM) low glucose (1 g L^–1^, Capricorn, Cat. No. DMEM-LPSTA) was utilized for cell line propagation. The medium was supplemented with 10% (v/v) fetal bovine serum (FBS, Capricorn, Cat. No. FBS-11A), 100 U mL^–1^ penicillin (Capricorn, Cat. No. PS-B), and 100 μg mL^–1^ streptomycin (Capricorn, Cat. No. PS-B). Phenol red-free DMEM (Gibco, Cat. No. A1443001) was used during live cell imaging. SYTO 59 Red Fluorescent Nucleic Acid Stain was purchased from Thermo Fisher Scientific.

Live cell imaging using CLSM was carried out to confirm the intracellular localization of the NPs. For this purpose, L929 cells were seeded into CELLview cell culture dishes (four compartments) at a concentration of 10^5^ cells per mL (500 μL per compartment, *n* = 3) in DMEM low glucose (1 g L^–1^) medium, supplemented with 10% (v/v) FBS, 100 U mL^–1^ penicillin, and 100 μg mL^–1^ streptomycin at 37 °C in a humidified 5% CO_2_ atmosphere. After 24 h of adherence, the cells were washed once with phosphate-buffered saline (PBS), and the medium was replenished. Subsequently, the investigated NPs were added into the wells at a final concentration of 300 μg mL^–1^ and incubated for 24 h. Following this, the medium was discarded, the cells were washed once with PBS, and then phenol red-free DMEM supplemented with SYTO 59 (1:1,000 stock dilution, 5 μM) was added to each well. The cells were incubated with the stain for 30 min, after which the staining solution was removed, and the cells were washed three times with the same DMEM and then kept in a fresh medium. The cells were then imaged with a Zeiss LSM880 Elyra (Carl Zeiss), while the confocal dishes were maintained in a microscope incubation system at 37 °C and 5% CO_2_. The pyrene-functionalized NPs and SYTO 59 channels were excited at 405 and 633 nm, respectively. Images from a region of interest of 148 × 148 μm were acquired using a 40× water-immersion objective (N.A. 1.2) with a pixel size of 80 nm.

## 3 Results and Discussion

### 3.1 Polymer Synthesis and Characterization

The first step of our research comprised the identification of polymerization conditions suitable for the PyOH-initiated ROP of the three monomers PheG, IleG, and ValG. Initially, kinetic studies were conducted to determine an appropriate [M]/[I]/[DBU]/[TU] ratio, monomer concentration, and reaction time. Based on our previous experiences using different initiators,^11,19^ a value of 100/1/1/10 for the [M]/[I]/[DBU]/[TU] ratio and a monomer concentration of 0.5 mol L^–1^ were selected. Previous studies have also shown that PEAs with such targeted molar masses are suitable for NP formulation.^1,2,23^ The ROP was carried out at room temperature in dichloromethane.

For the determination of the kinetic polymerization behavior, samples were taken from the reaction solutions at specific time intervals, quenched with an excess of benzoic acid, and analyzed by means of ^1^H NMR spectroscopy and SEC. The monomer conversion was determined based on the methine signal of the monomer ring at about 4 ppm, while signals from substituents whose integrals remained constant over the reaction progress were used as reference.

The data points of all three polymerizations exhibit a linear progression in the kinetic plot (Figure 1, left), indicating pseudo-first-order reaction kinetics with the chain growth as the rate-determining step. Also, the number-average molar mass (*M*_n_) obtained by SEC increased linearly with the monomer conversion (Figure 1, right). Thereby, the maximum conversion values in the considered reaction times were about 80 to 90%, while the dispersity values (*Đ*) did not exceed 1.15. The overlays of the SEC elugrams of all three polymerizations revealed a predominant absence of transesterifications, as monomodal, narrow, and symmetrical molar mass distributions were obtained at any time (Figure S1).

**Figure 1:**
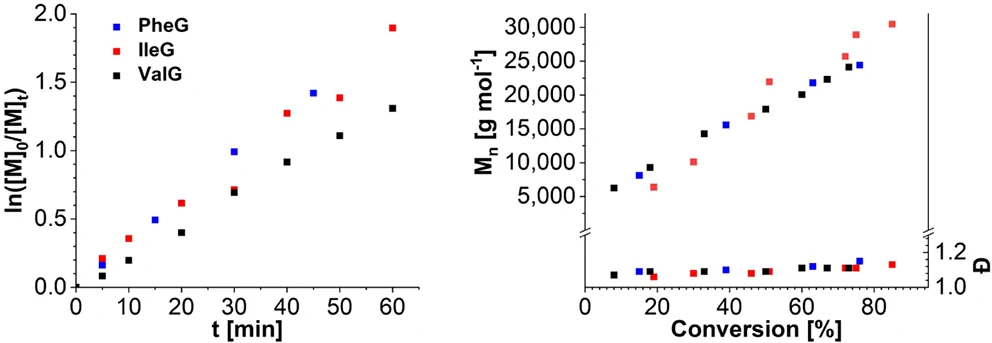
First-order kinetic plots (left) and evolution of *M*_n_ and *Đ* with the monomer conversion (right) for the PyOH-initiated polymerizations of PheG, IleG, and ValG in dichloromethane ([M]/[I]/[DBU]/[TU] = 100/1/1/10, [M]_0_ = 0.5 mol L^–1^, room temperature). *M*_n_ and *Đ* were obtained from SEC using PS calibration.

All analytical data collected from the kinetic studies indicated that the reaction conditions were controlled. Consequently, they were kept for the synthesis of **Py-PPheG**, **Py-PValG**, and **Py-PIleG** on a 1 g scale. For the calculation of the theoretical molar masses (*M*_n,theo_) of the resulting polymers, the monomer conversion values were determined by ^1^H NMR spectroscopy, as described in the kinetic studies. The obtained values of around 90% were only slightly higher than those observed during the kinetic studies and made us to expect DP_theo_ values of approximately 90, based on the used [M]/[I] ratio of 100/1 (Table 1). The *M*_n,theo_ values, thus, increased according to the molar masses of the respective repeating units.

**Table 1:** Selected Characterization Data of the Py-PEAs Determined by ^1^H NMR Spectroscopy and SEC

	Conv.^a^ [%]	DP_theo_ ^b^	*M*_n,theo_ ^b^ [g mol^–1^]	DP^c^	*M*_n_ ^d^ [g mol^–1^]	*Đ* ^d^
Py-PPheG	87	87	18,100	78	19,900	1.15
Py-PIleG	92	92	16,000	n.d.	25,400	1.12
Py-PValG	83	83	13,300	n.d.	19,200	1.14

a Determined from the ^1^H NMR spectra of the reaction solutions.

b Calculated from the conversion and the [M]/[I] ratio.

c Determined from the ^1^H NMR spectra of the purified polymers, calculated from the integrals of the methylene proton signal of the pyrene end group and substituent signals.

d Determined by SEC (DMAc, 0.21% (w/w) LiCl; RID; PS calibration).

Based on that consideration, the elution of **Py-PPheG** during SEC measurements would be expected at a lower elution volume than **Py-PIleG**, while **Py-PValG** should be detected at higher elution volumes. However, in the overlay of the SEC elugrams of the obtained polymers (Figure 2), the molar mass distribution of **Py-PPheG** was detected at roughly the same elution volume as **Py-PValG**. This suggests a denser coiling of the **Py-PPheG** polymer chains in the SEC eluent compared to the other Py-PEAs. This may be caused by additional attractive intramolecular interactions through π–π stacking of the phenyl rings of **Py-PPheG** in the rather polar eluent DMAc supplemented with LiCl. Furthermore, the determined molar masses, in particular those of **Py-PIleG** and **Py-PValG**, differ significantly from the theoretical values (Table 1). This is due to the use of PS as the calibration standard. The obtained *Đ* values vary between 1.12 and 1.15 and are, thus, similar to the values received in the kinetic studies, showing that the ROPs were well-repeatable.

**Figure 2:**
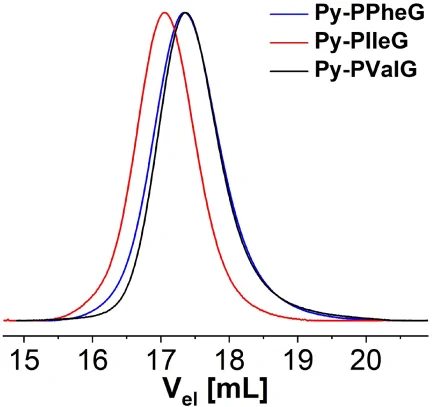
Overlay of the SEC elugrams (DMAc, 0.21% (w/w) LiCl; RID) of **Py-PPheG**, **Py-PIleG**, and **Py-PValG**.

In addition to the RID, a DAD was used for the SEC measurements. By overlaying the signals from the RID and DAD, a covalent attachment of the pyrene to the polymers was confirmed, since the molar mass distribution detected by the DAD was recorded at the same elution volume as the RID signal (Figure 3, Figures S2 and S3). In addition, the characteristic absorbance spectrum of the aromatic system of the pyrene α-end group was visible upon elution, thereby confirming the preservation of its chromophore.

**Figure 3:**
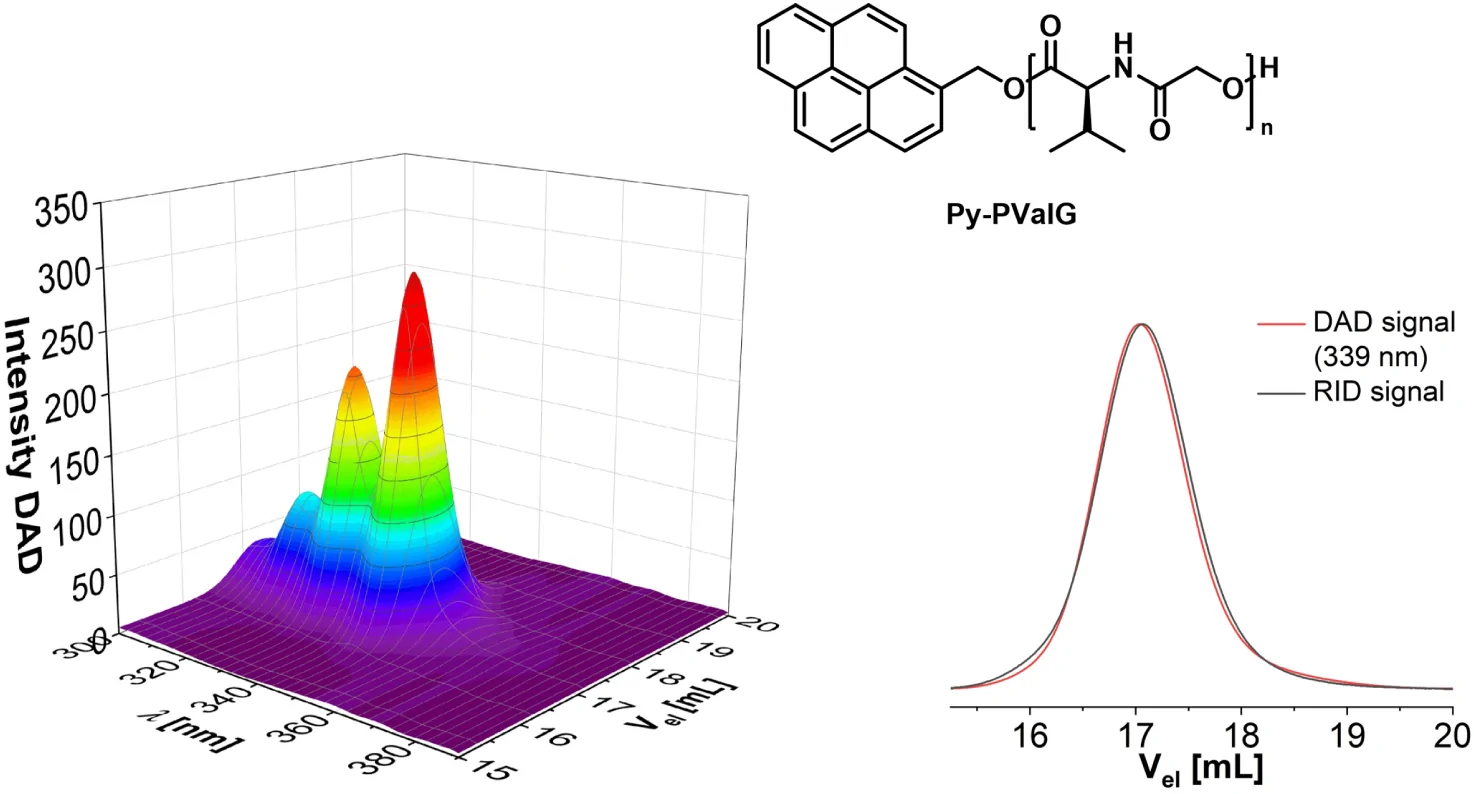
SEC analysis of **Py-PValG** (DMAc, 0.21% (w/w) LiCl; RID; DAD). Left: 3D plot depicting the absorbance spectrum of the pyrene end group during sample elution (DAD). Right: overlay of the SEC elugrams recorded by the RID (gray) and the DAD (red, 339 nm).

Moreover, also ^1^H NMR spectroscopy indicated the presence of the pyrene moiety. All observed signals were successfully assigned to the polymeric structures of **Py-PPheG**, **Py-PIleG**, and **Py-PValG** (Figures S4, S5, and S6). In addition to aromatic signals, the methylene proton signals of the pyrene α-end group between 5.8 and 6.0 ppm were detected. For **Py-PPheG**, a DP of around 80 was determined, which is in general accordance with the expected value (Table 1) and corresponds to a molar mass *M*_n,NMR_ of 16.2 kg mol^–1^. However, the splitting of the methylene proton signal in the spectra of **Py-PIleG** and **Py-PValG** made integration less accurate. We therefore refrained from estimating DP and *M*_n,NMR_ values from the ^1^H NMR spectroscopy in those cases.

The end-group fidelity was also investigated using MALDI-TOF MS. MALDI-TOF mass spectra of all polymers were recorded using DCTB or dithranol as the matrix and NaTFA as the doping salt (Figure S7). In general, the Py-PEAs could be ionized up to *m*/*z* ratios of 9000 and higher under the applied measurement conditions. **Py-PPheG** was best ionized with DCTB, even if milder conditions were used than those used in the measurement with dithranol. In contrast, **Py-PIleG** and **Py-PValG** were better ionized with a slightly more hydrophilic dithranol matrix.

Figure 4 displays an overview of the corresponding analytical results for **Py-PPheG**. The MALDI-TOF MS results of the other polymers were similar and are depicted in the Supporting Information.

**Figure 4:**
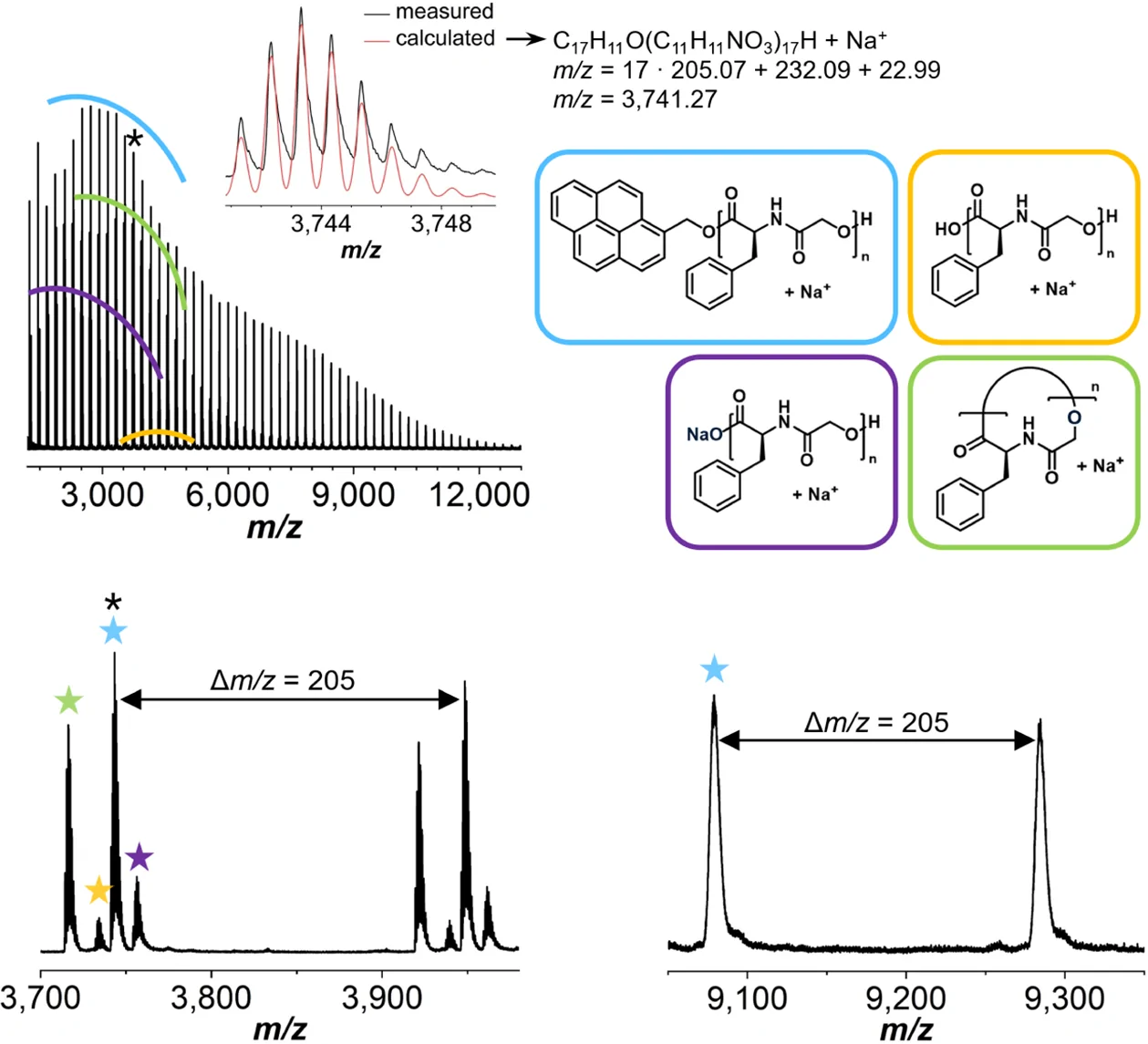
MALDI-TOF MS analysis of **Py-PPheG** (DCTB, NaTFA). Top left: full mass spectrum of **Py-PPheG** with highlighted distributions of the detected species and overlay of the measured and calculated isotopic pattern of the most abundant species. Top right: overview of the detected species. Bottom: zooms into the spectrum at different *m*/*z* ratios.

The spectrum of **Py-PPheG** shows that, despite the high molar mass of the polymer (*M*_n,theo_ = 18,100 g mol^–1^), intact chains of the desired polymer species (blue) were detected up to a high *m*/*z* ratio of 13,000. Furthermore, carboxy end-functionalized species (orange) and their sodium salts (purple) were detected. In principle, such structures can be formed through initiation by traces of water during polymerization. However, since the highest abundance of these species was observed at low *m*/*z* ratios, cleavage of the pyrene end group and fragmentation as a result of the measurement conditions are more likely to be the cause. In addition, cyclic PEA species were observed (green), which were presumably formed by intramolecular transesterification during the ROP. A zoom into the spectrum shows that the *m*/*z* difference between two monoisotopic peaks is 205, which equals the exact mass of the repeating unit of **Py-PPheG**. The final proof of the structure of **Py-PPheG** and the successful functionalization with pyrene was obtained by an overlay of the calculated and measured isotopic patterns.

In summary, the ROP of three different morpholine-2,5-diones was efficiently accomplished using PyOH as an initiator. In fact, polymerization conditions could be directly transferred from the established initiator benzyl alcohol without requiring additional optimization. Our next step was to investigate whether this simple change in the initiator could offer additional benefits in the application of the obtained Py-PEAs.

### 3.2 Nanoparticle Formation

PValG-, PIleG-, and PPheG-based NPs have recently been applied for the intracellular delivery of the anti-inflammatory drugs 6BIGOE, MF-15, BRP-201, and BRP-187.^1,2,23^ Following a modified in-house protocol established in our laboratories for the formulation of PEAs without pyrene end groups,^1,2^ the synthesized Py-PEAs were used to form NPs via nanoprecipitation (Table S1). Initially, three NP batches were prepared per polymer by nanoprecipitation from THF into water without the use of any stabilizer. The *d*_h_ determined by DLS ranged from 97 to 111 nm on average, with PDI values of approximately 0.15 (Table 2). The NPs revealed neutral ζ. Despite that, characterization by SEM (Figure 5) revealed uniformly sized and spherical particles with no signs of aggregation. Because SEM measurements were performed in a dried state, mean diameters of the NPs tended to be lower than the *d*_h_ values from DLS (Figure S14). Overall, the results revealed that the Py-PEAs are suitable for the formulation of NPs even without the use of a stabilizer.

**Figure 5:**
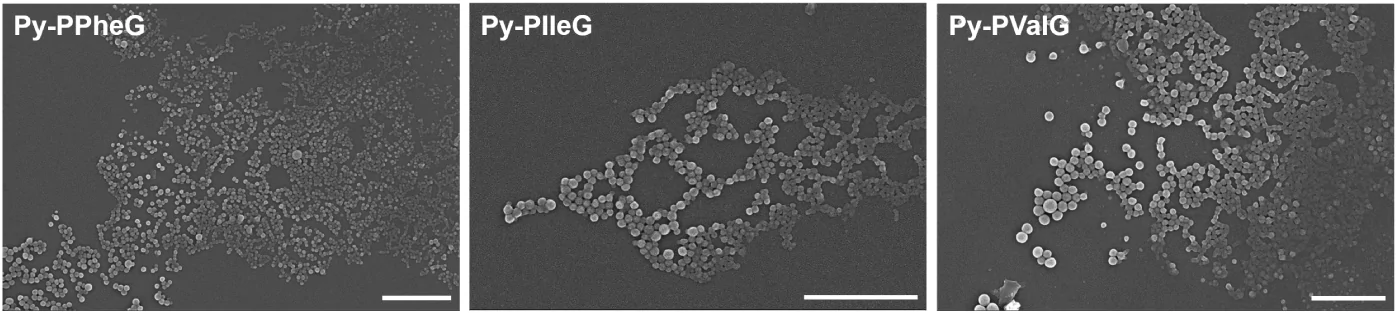
SEM images of the NPs based on **Py-PPheG**, **Py-PIleG**, and **Py-PValG** formulated without PVA (scale bar equals 1 μm).

**Table 2:** Overview of the Characteristics of NPs Prepared under Different Formulation Conditions as Determined by DLS and ELS

	THF/water^a^			Acetonitrile/water with PVA^b^		
NP	Py-PPheG	Py-PIleG	Py-PValG	Py-PPheG	Py-PIleG	Py-PValG
*d*_h_ [nm]	97 ± 8	111 ± 6	106 ± 0	163	158	160
PDI	0.15 ± 0.01	0.16 ± 0.02	0.15 ± 0.02	0.07	0.04	0.08
ζ [mV]	0 ± 0	–2 ± 1	0 ± 0	–25	–25	–25
Yield [%]	-	-	-	78	72	72

a Formulations performed using a polymer concentration of 2 mg mL^–1^ in THF (*n* = 3); particles were not purified; therefore, no yield is given; characterization after solvent evaporation.

b Formulation performed using a polymer concentration of 10 mg mL^–1^ in acetonitrile with 0.3% (w/v) PVA in water (*n* = 1); particles were purified; characterization after purification.

Previous studies demonstrated the successful formulation of PEAs into NPs.^1,2^ In the present work, we confirmed the robustness of the formulation and revealed that the pyrene end group of the Py-PEAs does not interfere with the NP formulation or their characteristics.

As no sufficiently reproducible fluorescence spectra could be recorded from the aqueous NP suspensions, NPs were prepared with an optimized protocol to increase the stability of the formulations. In this procedure, PVA was used as the stabilizer and acetonitrile as the solvent, with a polymer concentration of 10 mg mL^–1^. Those NPs tended to be larger, featuring *d*_h_ values around 160 nm, decreased PDI values below 0.08, and a negative ζ of −25 mV or lower (Table 2). In particular, the latter finding is consistent with the described PVA-mediated stabilization effects.^47,48^

### 3.3 Fluorescence Spectroscopy: Pyrene as a Label for Nanoparticle Degradation

The Py-PEAs as well as their NPs were characterized by fluorescence spectroscopy to explore the added value of the pyrene end group as a fluorescent label to monitor NP degradation. In the so-called 3:1 method, the change in the intensity ratio of two specific bands in the fluorescence spectrum of pyrene caused by the change of the molecular environment is used. According to Kalyanasundaram and Thomas, the bands at around 373 nm (band I) and 383 nm (band III) are used for this purpose.^37^ The ratio of the corresponding intensities (*I*_III_/*I*_I_) provides a measure of the properties of the molecular environment of pyrene. In general, a low value indicates a polar and hydrophilic environment, while higher values hint at a decreased polarity and an increased hydrophobicity. However, the change of *I*_III_/*I*_I_ may be sensitive to numerous environmental factors, including local polarity, aggregation, polymer chain mobility, and NP disassembly. We hence applied DLS as a complementary and well-established method to monitor NP degradation.

Figure 6 (left) depicts the fluorescence spectra of the Py-PEAs measured in THF. Since the pyrene moiety is covalently attached to the polymers, the bands were slightly shifted to higher wavelengths compared to those of free pyrene. The intensity values for band I were taken at 376 nm and for band III at 387 nm, yielding *I*_III_/*I*_I_ ratios of approximately 0.75 (Table 3). In comparison, the literature value of free pyrene in THF is 0.83.^37^ Consequently, the pyrene end group experiences a more polar molecular environment in the polymer solution than in pure THF, most likely due to the fact that the polymer structure contains polar amide bonds.

**Figure 6:**
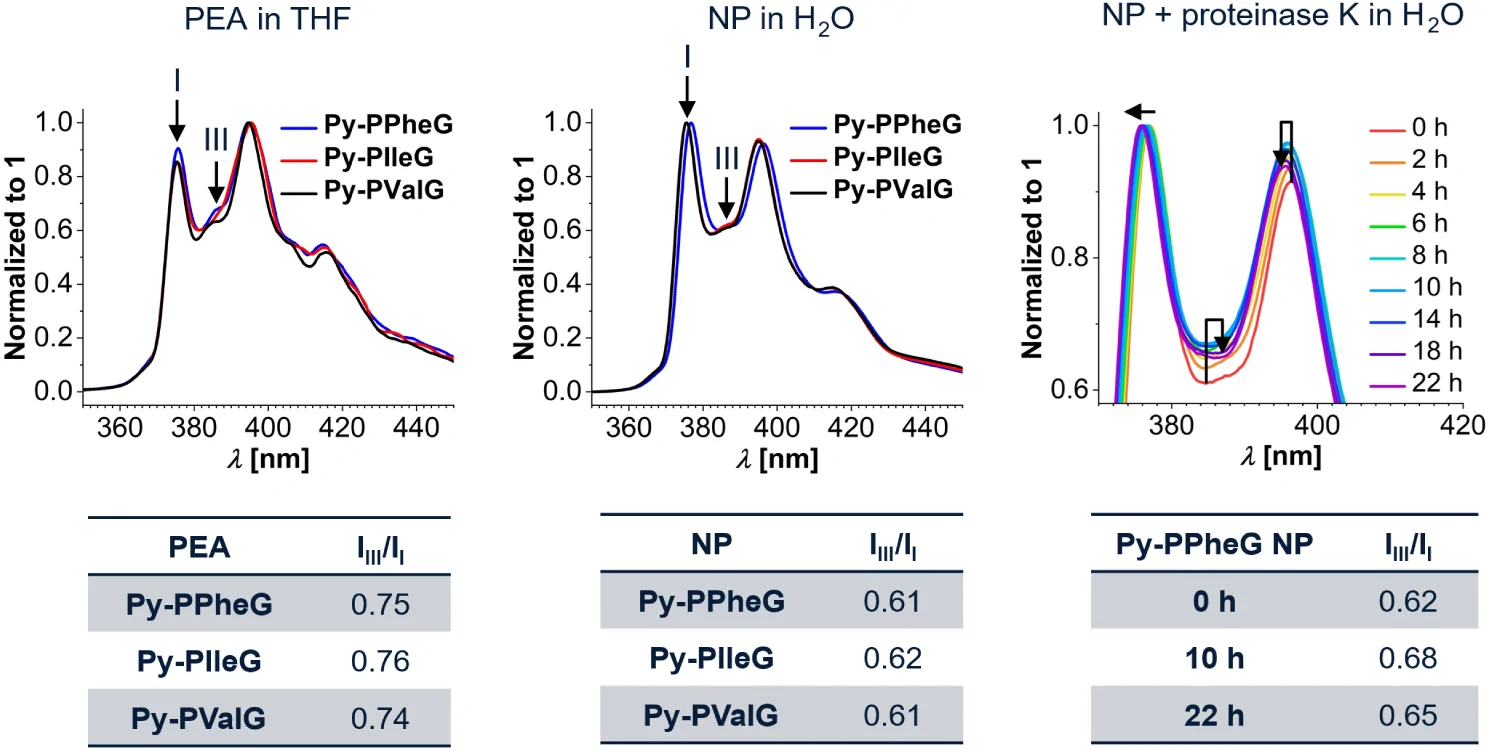
Fluorescence spectroscopy of the Py-PEAs indicating the intensities and shifts of bands I and III in different media (excitation at 339 nm). *I*_III_/*I*_I_ ratios of band III at 387 nm and band I at 376 nm are listed at the bottom. Left: fluorescence spectra of the Py-PEAs at 25 °C in THF (*c* = 0.01 mg mL^–1^). Middle: fluorescence spectra of the corresponding NPs at 25 °C in purified water (*c*_NP_ = 6.5 μg mL^–1^). Right: zoom into the fluorescence spectra recorded during the degradation study of **Py-PPheG** NPs using proteinase K (PK) in purified water (*c*_NP_ = 6.5 μg mL^–1^, *c*_PK_ = 167 μg mL^–1^, 37 °C). The arrows indicate the band shifts over time.

**Table 3:** Overview of the Intensity Ratios *I*_III_/*I*_I_ of Pyrene and Py-PEAs in Water, THF, or Aqueous Proteinase K Solution^a^

			NP degradation studies	
	Pyrene	Py-PEA	Max	End
H_2_O	0.63 ^37^	0.61–0.62^b^	-	0.69^c^
THF	0.83 ^37^	0.74–0.76^d^	-	-
Aq. proteinase K	-	0.61–0.62^e^	0.67–0.68^e^	0.65^e^

a Maximum and end values observed during the degradation experiments of the NPs are also indicated.

b Obtained by fluorescence spectroscopy of the NPs of **Py-PPheG**, **Py-PIleG**, and **Py-PValG** (*c*_NP_ = 6.5 μg mL^–1^, 25 °C).

c Obtained by degradation experiments using **Py-PPheG**-based NPs in 0.002 M NaOH (*c*_NP_ = 6.8 μg mL^–1^, 25 °C).

d Obtained by fluorescence spectroscopy of the polymers (*c* = 0.01 mg mL^–1^, 25 °C).

e Obtained during the degradation kinetic studies of the NPs based on **Py-PPheG**, **Py-PIleG**, and **Py-PValG** (*c*_NP_ = 6.5 μg mL^–1^, 37 °C).

Furthermore, fluorescence spectra of the corresponding NPs in purified water were measured (Figure 6, middle). As PEAs are usually suitable for the encapsulation of hydrophobic drugs,^1,2,23^ the pyrene end group is expected to be embedded in the core of the NP. Thus, we expected a higher *I*_III_/*I*_I_ value compared with that of free pyrene in water (*I*_III_/*I*_I_ = 0.63).^37^ In contrast, values from 0.61 to 0.62 were obtained for all three NP types (Table 3). This hints at a more hydrophilic and polar nature of the Py-PEAs than expected and can be explained by the amide and ester bonds, which are also able to form hydrogen bonds. On the other hand, water diffusion into the NPs or the location of pyrene moieties on the NP surface might also contribute to the observations. A falsification of the value due to bleeding of the dye can be excluded, as the covalent attachment of the pyrene to the polymers was consistently confirmed by all polymer characterization data.

We next explored changes in the fluorescence spectra upon NP degradation to examine the behavior of the pyrene end group as a potential sensor. An initial degradation test was performed in a 0.002 M aqueous NaOH solution in triplicate (*c*_NP_ = 6.8 μg mL^–1^). The samples were directly investigated by fluorescence spectroscopy, stored for 24 h at 37 °C, and then remeasured. In addition, blanks, i.e., samples without NaOH, were investigated to ensure that NPs remained stable until the measurement of the *t*_0h_ sample. As shown in Figure S15, the same spectrum with an *I*_III_/*I*_I_ of 0.61 was obtained for all three *t*_0h_ samples as well as for the blank samples. The similarity of the spectra confirmed that the addition of small amounts of NaOH did not affect the apparent polarity in the surroundings of the pyrene moiety during the few minutes required to record the spectra. Surprisingly, the *I*_III_/*I*_I_ value was consistently elevated after storage at 37 °C for 24 h. The obtained value of 0.69 (Table 3) points toward a less hydrophilic environment of the fluorescent probe after NP degradation. This was surprising, as the release of pyrene from an NP into an aqueous environment was expected to result in a decreased value. However, the NP degradation was confirmed by DLS, and the high polarity of the Py-PEA NPs might explain the observation.

The enzymatic NP hydrolysis is of higher interest regarding the application of NPs for drug delivery. Hence, degradation experiments using proteinase K and **Py-PPheG** NPs were carried out in a similar fashion (Figure S16). To ensure a reasonable time frame for the experiment,^2^ we selected an NP-to-proteinase K mass ratio of 1:25. Again, the initial intensity ratios were 0.61 or 0.62 and increased to 0.65 after 4 h and to 0.69 after 24 h. The fact that the same tendencies were observed during the degradation with NaOH proves that the presence of proteinase K does not influence the values.

Performing the experiments in triplicate using **Py-PPheG** (Figure S16) confirmed the robustness and repeatability of the method. We next focused on more detailed proteinase K-catalyzed degradation studies using all three Py-PEAs, i.e., **Py-PPheG**, **Py-PIleG**, and **Py-PValG**. Therefore, NP dispersions with an NP-to-enzyme mass ratio of 1:26 were kept at 37 °C, and fluorescence spectra as well as DLS correlograms were recorded at regular time intervals.

Figure S17 presents the overlays of the fluorescence spectra for all three Py-PEA NP batches, revealing band shifts and intensity changes over time in any case. In addition, zoomed-in overlays of the spectra for **Py-PPheG** in Figure S18 and Figure 6 exemplarily illustrate the spectral changes over time in more detail. Specifically, small shifts of the maxima of band I and the band at 396 nm were visible with increasing time. Furthermore, the intensities of the latter and band III gradually increased until maximum intensities in relation to those of band I were reached. Subsequently, a decrease in the intensities at the corresponding wavelengths was observed until the end of the degradation experiment.

The changed band intensities over the course of the degradation are reflected in the *I*_III_/*I*_I_ ratios (Figure 7). In general, an increase in the ratios was observed until a maximum was reached. Afterward, the values decreased again, and the plots flattened out. The similar *I*_III_/*I*_I_ values of the different Py-PEA NPs, 0.61 or 0.62 at the beginning of the experiments, indicate similar polarity of the corresponding NPs. The maximum *I*_III_/*I*_I_ values as indicated in Figure 7 were reached at varying degradation times depending on the Py-PEA. The slowest increase was observed for the **Py-PPheG**-based NPs, where the maximum was reached after 9.5 h. The highest *I*_III_/*I*_I_ value was observed after 6 h for **Py-PIleG** NPs. The *I*_III_/*I*_I_ plot of the **Py-PValG** NPs featured the steepest increase. Here, the maximum was reached after just 1 h. We suspected that these time points indicated complete NP degradation and, thus, closely inspected the DLS data (Figure 7, middle and bottom). The normalized count rate only dropped to around 30% at the corresponding times, pointing to incomplete degradation. On the other hand, increasing noise was observed in the correlation functions from these time points onward, which means that reliable sizes can no longer be derived from DLS. Throughout the further course of the experiment, the scattering of the DLS correlation functions even increased. Hence, the reported NP sizes must be taken with more than a grain of salt. Yet, the apparent increase in size toward the end of the NP degradation may indicate an aggregation of degradation products including pyrene. This process could explain the decreasing *I*_III_/*I*_I_ values observed by fluorescence spectroscopy after reaching maximum values and was confirmed by SEM imaging of degraded **Py-PValG** NPs (Figures S20 and S21). Loose aggregates were detected only occasionally, whereas no particles were found on the majority of images received. The aggregation seemed especially enhanced in the case of the **Py-PPheG** NPs, which may be due to additional interactions such as the π–π stacking between the aromatic systems of pyrene and the benzyl substituents of the degradation products. The flattening of the generated plots using the fluorescence spectroscopic data could then be interpreted as the establishment of an aggregation equilibrium. The data from degraded samples found by SEM were consistent with MALDI-TOF MS (Figure S22). Under identical measurement conditions, polymer signals were rarely detected, which contrasts with a sample measured prior to degradation. Depending on the position of the laser on the spot, either no signal or low-intensity signals were found. No signals were detected at higher *m*/*z* values, confirming the hydrolysis of ester moieties in the polymer chains.

**Figure 7:**
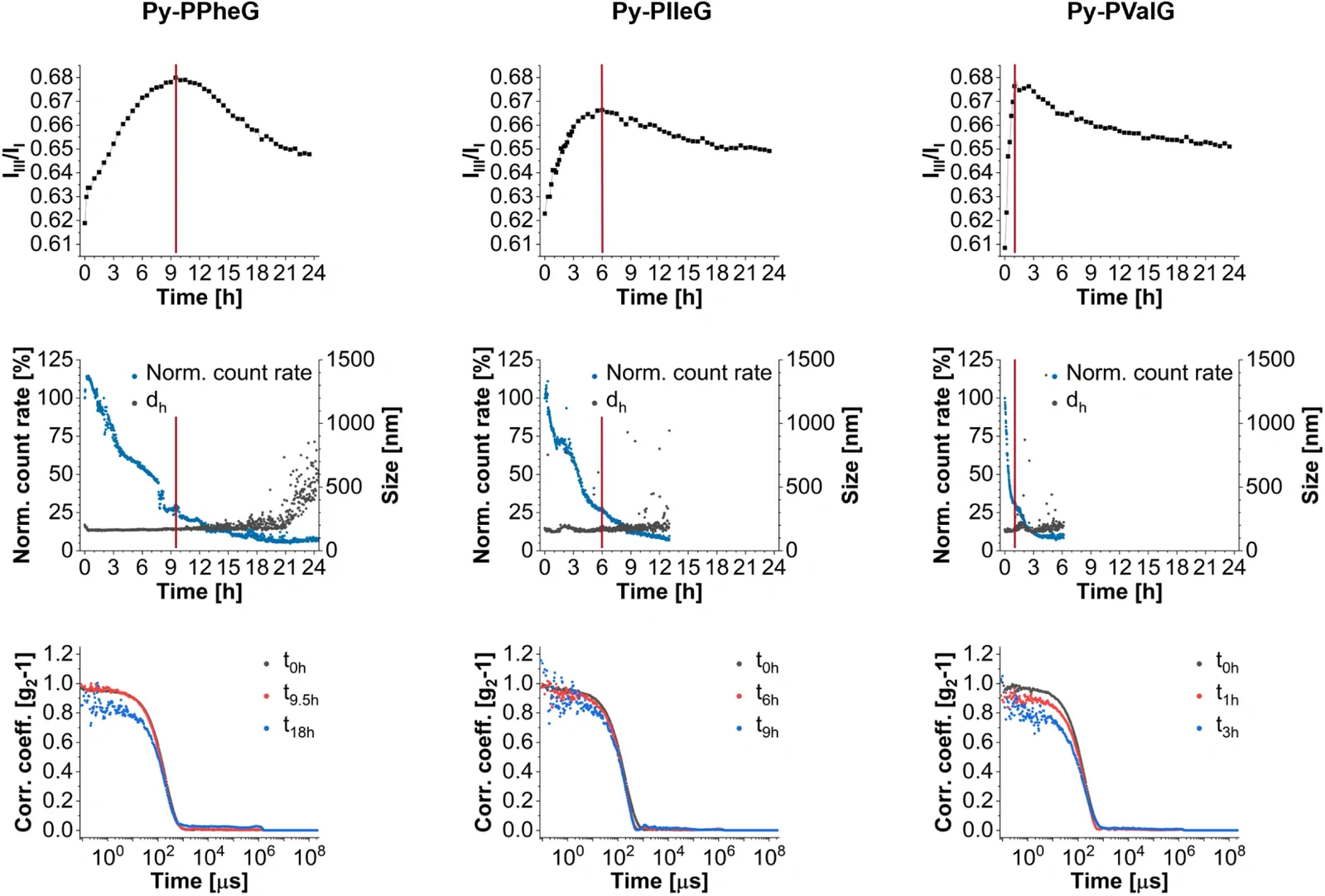
Proteinase K-mediated NP degradation studies in purified water (*c*_NP_ = 6.5 μg mL^–1^, *c*_PK_ = 167 μg mL^–1^, 37 °C). Samples were analyzed by means of fluorescence spectroscopy and DLS throughout the course of the degradation. Top: intensity ratios (*I*) of band III (387 nm) and band I (approximately 376 nm) received from fluorescence spectroscopy against the degradation time. Middle: normalized count rate and NP size determined by DLS against time. Bottom: selected DLS correlation functions (gray: *t*_0h_, red: time of maximum *I*_III_/*I*_I_ as indicated in the top row, blue: exemplary time point with normalized count rate below 10%).

In summary, the data demonstrated that monitoring the degradation of Py-PEA-based NPs by fluorescence spectroscopy is possible and represents an alternative to conventional and most commonly used monitoring by DLS. Clear differences in the degradation rates were discernible with either technique. NPs made of PEA featuring sterically demanding substituents degraded more slowly: the benzyl-substituted **Py-PPheG** exceeded the degradation time of *sec*-butyl-substituted **Py-PIleG**, and the *iso*-propyl-substituted **Py-PValG** degraded the fastest.

These trends in the degradation behavior have already been observed in a previous study, in which nanoparticulate systems based on PEAs with a benzyl α-end group were investigated for the delivery of anti-inflammatory drugs.^2^ Apparently, no change in the degradation behavior of the corresponding NPs was found as a result of the change of the α-end group. Yet, additional information was accessible through the use of the pyrene moiety.

### 3.4 Cellular Uptake: Pyrene as a Label for CLSM

In addition to tracking NP degradation, the incorporation of a fluorescence-active initiator, such as a pyrene derivative, offered further practical advantages. Although pyrene requires excitation at relatively low wavelengths that are less than ideal for live cell imaging, modern CLSM systems are technically equipped with suitable lasers that can accommodate its detection.^32,49,50^

To demonstrate that the investigated NPs can also be used for efficient intracellular delivery, a cellular internalization study was performed. The L929 cell line was chosen as a standard fibroblast model for these assays, and CLSM was employed to track the internalization of the pyrene-functionalized Py-PEA NPs. Internalization was assessed after a 24 h incubation period with the NPs at a concentration of 300 μg mL^–1^ (Figure 8). To facilitate efficient detection, the cell nuclei were stained with SYTO 59 (red fluorescence), while the NPs were visualized as a white signal. The results revealed that Py-PEA NPs were readily internalized by the cells within 24 h, as evidenced by the increased fluorescence intensity compared to untreated control cells. We attempted a quantification of the cellular uptake by determining the mean fluorescence intensity (MFI) of the individual cells using three images per sample. The resulting values are expressed as x-fold changes in MFI compared to the control cells without NPs, after correction by the fluorescence intensity of the individual NP suspensions at 415 nm (Figure S23). The quantitative comparison of the three Py-PEA nanoparticles is confined due to the relatively high variance in fluorescence intensity between the individual cells, even within one image of a sample, leading to pronounced standard deviations. Nevertheless, this is often observed for biological samples. While the attached pyrene moiety is certainly able to serve as a label for preliminary cellular uptake studies, one should indeed switch to more specialized dyes as soon as more detailed *in vitro* studies come into focus.

**Figure 8:**
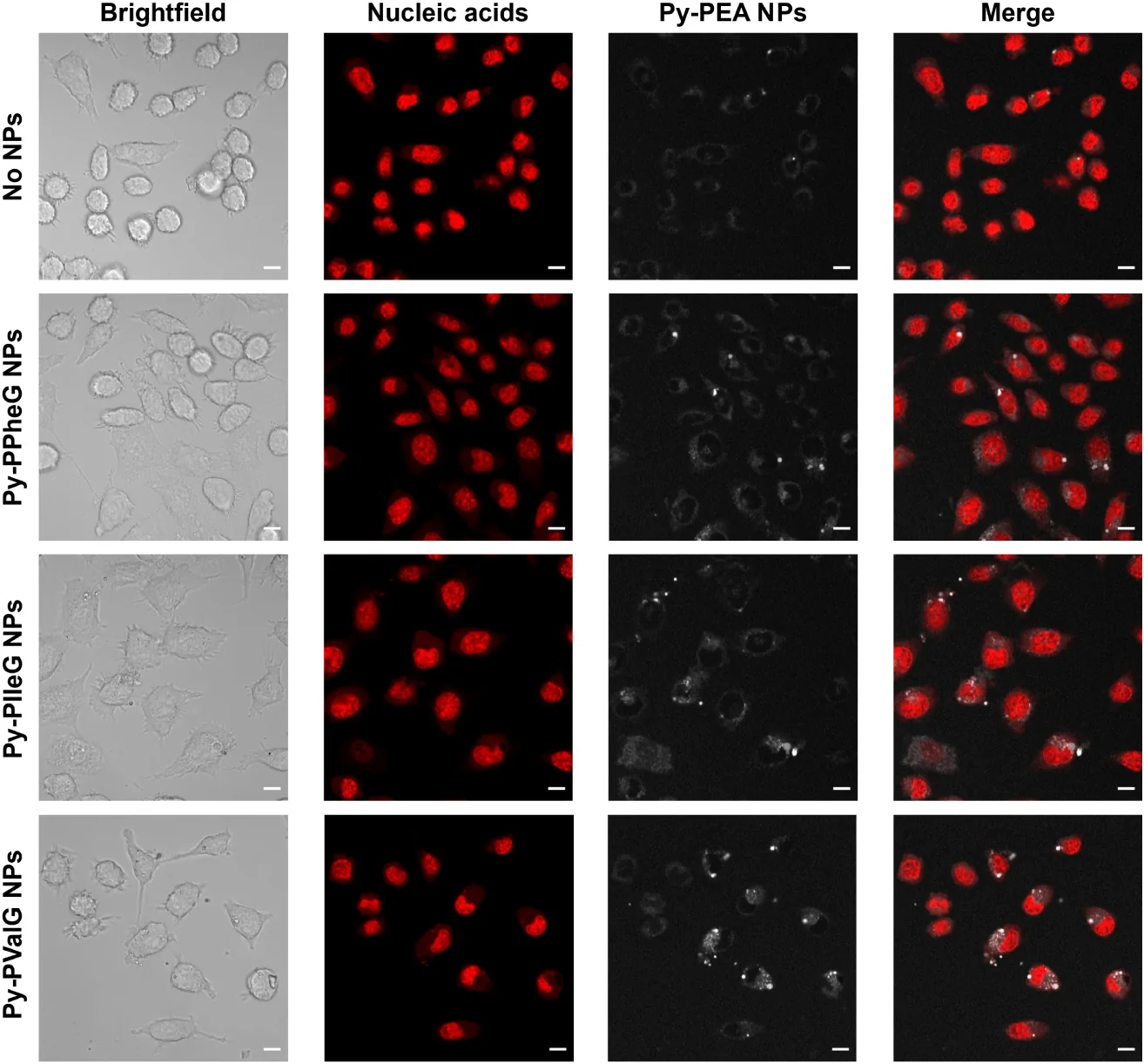
Cellular internalization of Py-PEA NPs by L929 cells. Cells were incubated with 300 μg mL^–1^ of the studied NPs for 24 h. Subsequently, the cells were washed, and the nucleus was stained with SYTO 59 as per the manufacturer’s protocol. Images were acquired using CLSM (red: SYTO 59, white: Py-PEA NPs; the scale bar equals 10 μm).

## 4 Conclusion

In this study, the successful introduction of a fluorescence-active pyrene α-end group in PEAs was demonstrated. For this purpose, the three morpholine-2,5-diones PheG, IleG, and ValG were subjected to ROP at room temperature. PyOH was used as an initiator, as well as a binary catalyst system consisting of DBU and TU. The kinetic studies indicated controlled reaction conditions within the considered reaction times, with maximum monomer conversion values of up to almost 90%, whereby narrow and monomodal molar mass distributions were obtained. The synthesized polymers **Py-PPheG**, **Py-PIleG**, and **Py-PValG** were characterized in detail using ^1^H NMR spectroscopy, RID and DAD SEC, MALDI-TOF MS, and fluorescence spectroscopy. A covalent attachment of the pyrene to the well-defined PEAs was confirmed. The polymers were subsequently used to formulate NPs by nanoprecipitation. These were analyzed by DLS and SEM imaging, where spherical particles with a uniform size distribution were revealed. The pyrene-functionalized NPs were then subjected to degradation studies using proteinase K, which were monitored by fluorescence spectroscopy. The obtained results were confirmed by parallel DLS measurements. To the best of our knowledge, this was the first demonstration of monitoring NP degradation with covalently bound fluorescence-active pyrene, applying fluorescence spectroscopy and the so-called 3:1 method. Using this analytical method, conclusions were drawn about the different degradation rates of **Py-PPheG**, **Py-PIleG**, or **Py-PValG** NPs and about secondary processes arising from the degradation, which are in line with previous studies. Furthermore, the pyrene-functionalized NPs were successfully internalized into L929 cells, which was visualized by CLSM. This highlights the added value of introducing a fluorescence-active compound such as pyrene into the polymer structure during polymerization. Thus, even more information can be collected at an early stage of the development of nanoparticulate drug delivery systems in order to identify the best polymer candidates for the specific planned application without additional synthesis effort through postpolymerization modifications.

## Supplementary Material



## Notes

Detailed formulation parameters and NP characteristics, SEC elugrams recorded during the kinetic studies, SEC elugrams of **Py-PPheG** and **Py-PIleG**, ^1^H NMR spectra of all polymers, additional MALDI-TOF mass spectra, size distributions from DLS, evaluation of the NP diameters from the SEM images, additional fluorescence spectra, SEM images and MALDI-TOF mass spectra of degraded NP samples, and quantification of CLSM data (PDF)
