# Supplementary material for: Benefits of Pyrenemethanol as an Initiator for the ROP of Morpholine-2,5-Diones: Fluorescence Probing of Nanoparticle Degradation and Cellular Uptake
Source: ACS Polym Au. 2026 Jul 8;6(5):1363–75. doi: 10.1021/acspolymersau.6c00097 (PMC13673884; doi:10.1021/acspolymersau.6c00097)
Supplement: Supplementary file 1 [file lg6c00097_si_001.pdf]

Supporting Information

Benefits of pyrenemethanol as an initiator for the ROP of morpholine-2,5-diones: Fluorescence probing of nanoparticle degradation and cellular uptake

Johanna Schreiber,[1,2] Lea C. Klepsch,[1,2] Jana Ismail,[1,2] David Pretzel,[1,2] Antje Vollrath,[1,2] Christine Weber,[1,2] Ulrich S. Schubert[1,2,]*

[1]Laboratory of Organic and Macromolecular Chemistry (IOMC), Friedrich Schiller University Jena, Humboldtstr. 10, 07743 Jena, Germany

[2]Jena Center for Soft Matter (JCSM), Friedrich Schiller University Jena, Philosophenweg 7, 07743 Jena, Germany

Table S1. Formulation parameters for the nanoprecipitation of the Py-PEA and NP characteristics.

Sample		$c_{Polymer}$[d)] [mg mL^{-1}]	solvent	S/W[e)]	PVA content[f)]	d_h[g)] [nm]	PDI[g)]	ζ in water[h)] [mV]	Yield[i)] [%]
**Py-PPheG**	w/o PVA[a)]	2	THF	1:6	0	97 ± 8	0.15 ± 0.01	0 ± 0	-
**Py-PIleG**	w/o PVA[a)]	2	THF	1:6	0	111 ± 6	0.16 ± 0.02	−2 ± 1	-
**Py-PValG**	w/o PVA[a)]	2	THF	1:6	0	106 ± 0	0.15 ± 0.02	0 ± 0	-
**Py-PPheG**	w PVA[b)]	10	Acetonitrile	1:8	0.3	150 ± 4	0.06 ± 0.01	−30 ± 0	74.3 ± 0.08
**Py-PPheG**	w PVA[c)]	10	Acetonitrile	1:8	0.3	163	0.07	−25	77.7
**Py-PIleG**	w PVA[c)]	10	Acetonitrile	1:8	0.3	158	0.04	−25	71.8
**Py-PValG**	w PVA[c)]	10	Acetonitrile	1:8	0.3	160	0.08	−25	71.6

[a)] Formulation performed with n = 3, particles were not purified, characterization after solvent evaporation, size distributions depicted in Figure S10 and S11. [b)] Formulation performed with n = 3, particles were purified, characterization after purification, size distributions depicted in Figure S12. [c)] Formulation performed with n = 1, particles were purified, characterization after purification, size distributions depicted in Figure S13. [d)] Polymer concentration in organic solvent. [e)] Solvent to water ratio. [f)] PVA content in the water phase in % (w/v). [g)] Determined by DLS. [h)] Determined by ELS. [i)] Yield after purification determined by dividing the actual obtained particle mass by the theoretically possible particle mass multiplied by 100.

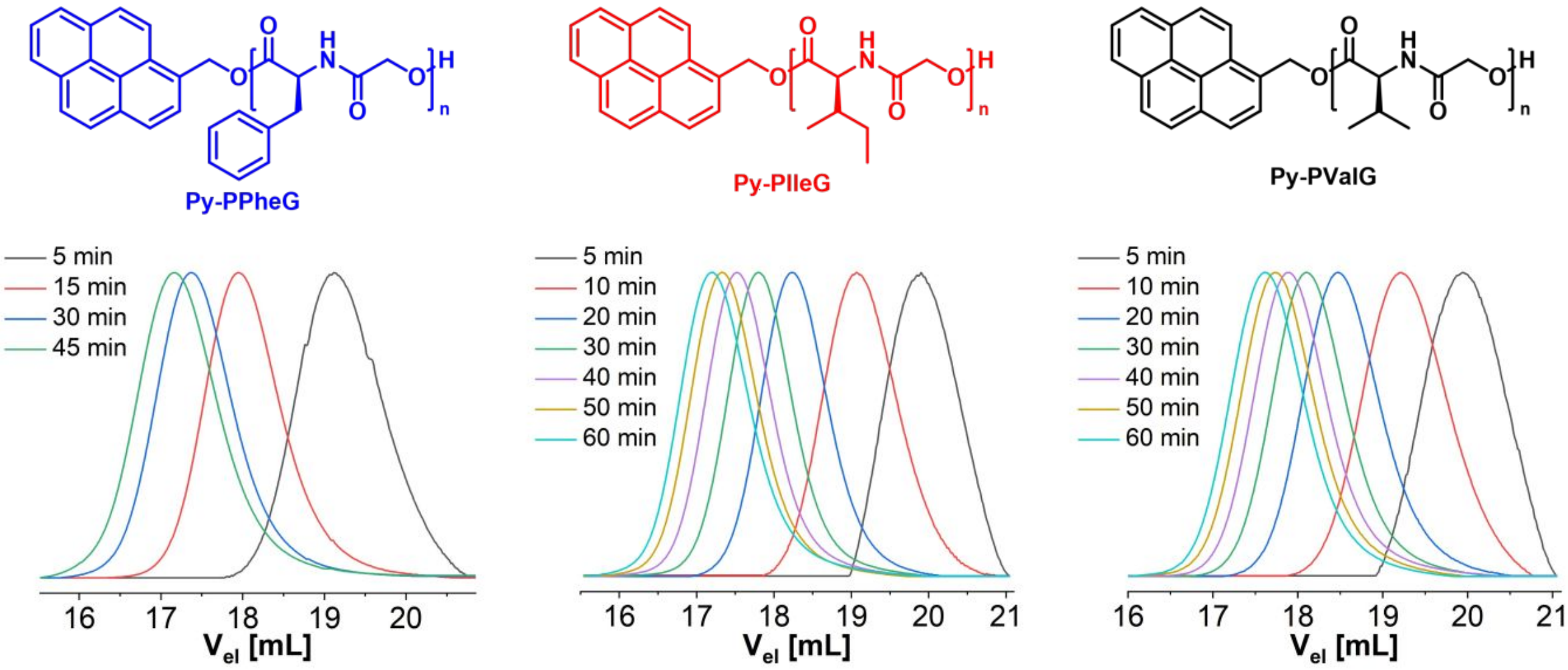

Figure S1. SEC elugrams (DMAc, 0.21% (w/w) LiCl; RID) obtained during the kinetic studies of the PyOH-initiated polymerizations of PheG, IleG and ValG in dichloromethane ([M]/[I]/[DBU]/[TU] = 100/1/1/10, $[M]_0$ = 0.5 mol L^{-1}, room temperature).

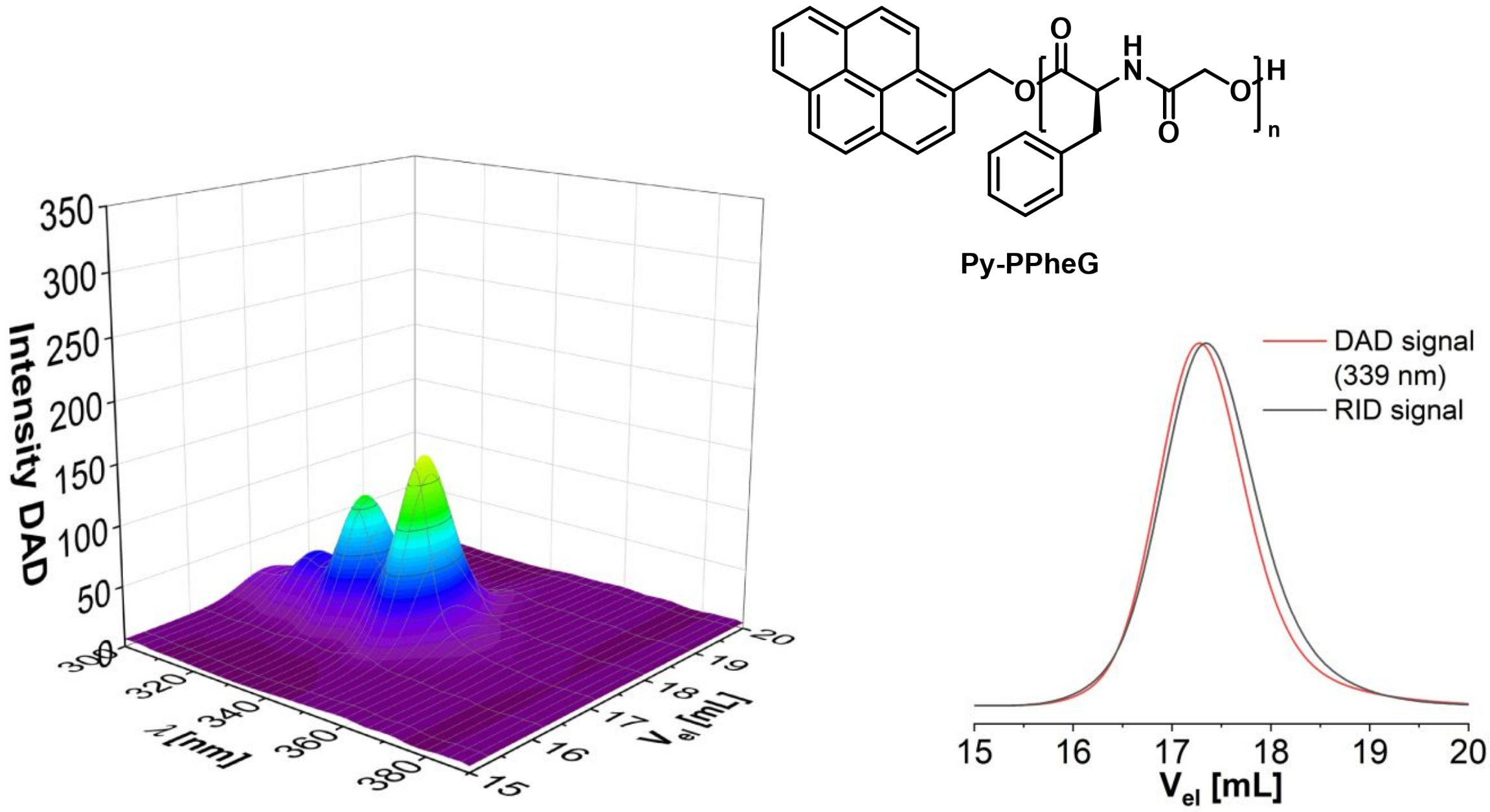

Figure S2. SEC analysis of **Py-PPheG** (DMAc, 0.21% (w/w) LiCl; RID; DAD). Left: 3D Plot depicting the absorbance spectrum of the pyrene end group during sample elution (DAD). Right: Overlay of the SEC elugrams recorded by the RID (gray) and the DAD (339 nm, red).

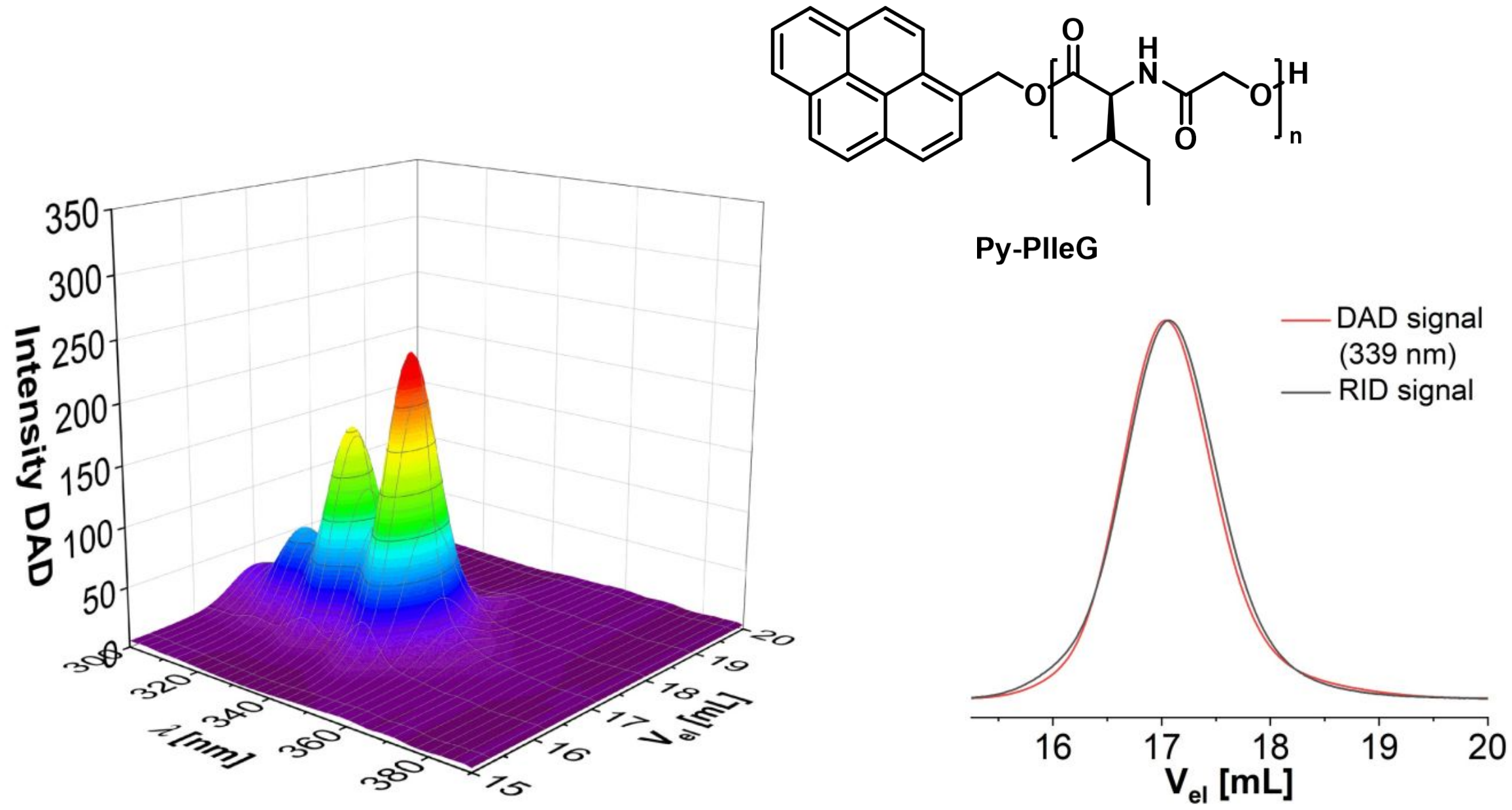

Figure S3. SEC analysis of **Py-PIleG** (DMAc, 0.21% (w/w) LiCl; RID; DAD). Left: 3D Plot depicting the absorbance spectrum of the pyrene end group during sample elution (DAD). Right: Overlay of the SEC elugrams recorded by the RID (gray) and the DAD (red, 339 nm).

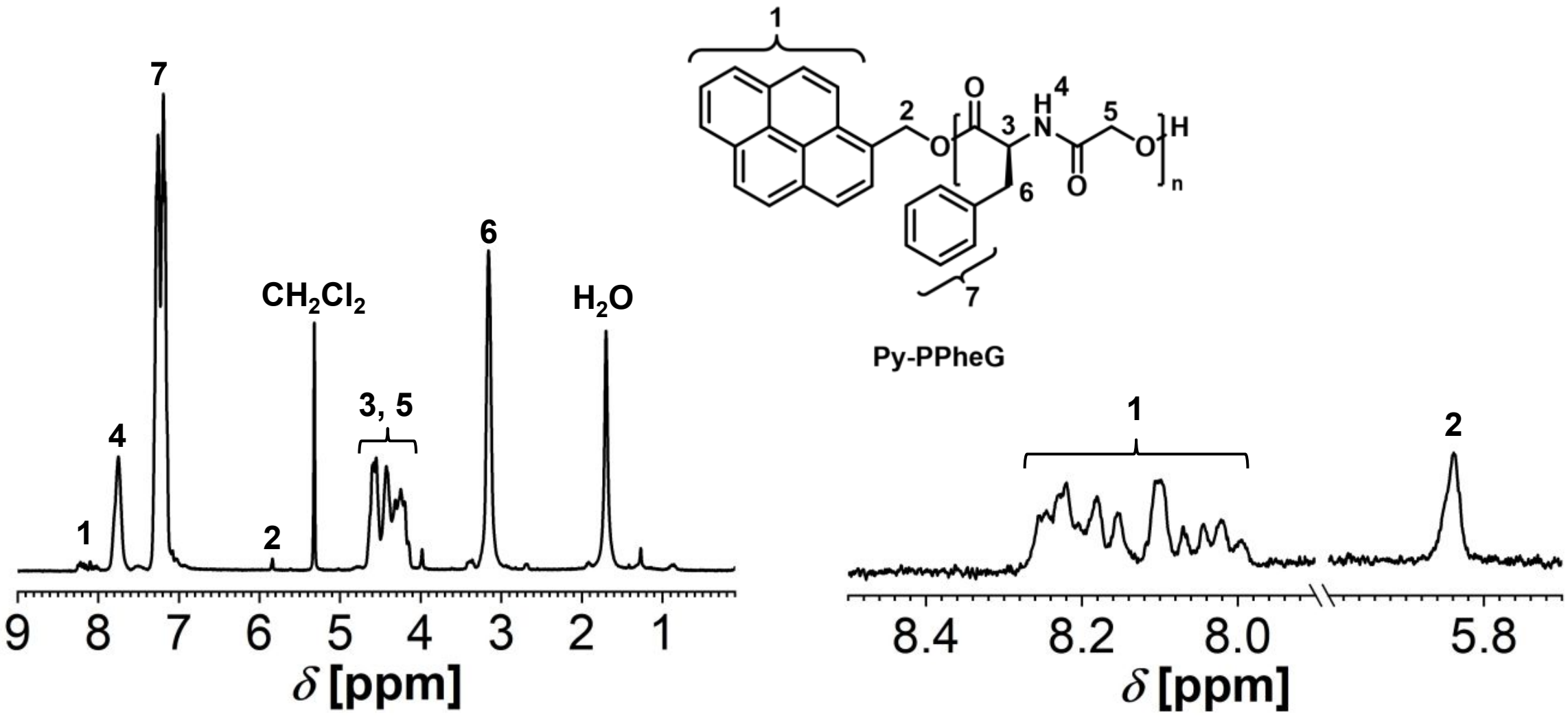

Figure S4. ^1H NMR spectrum (300 MHz, CD_2Cl_2) of **Py-PPheG** and zoom into the region of the α-end group signals.

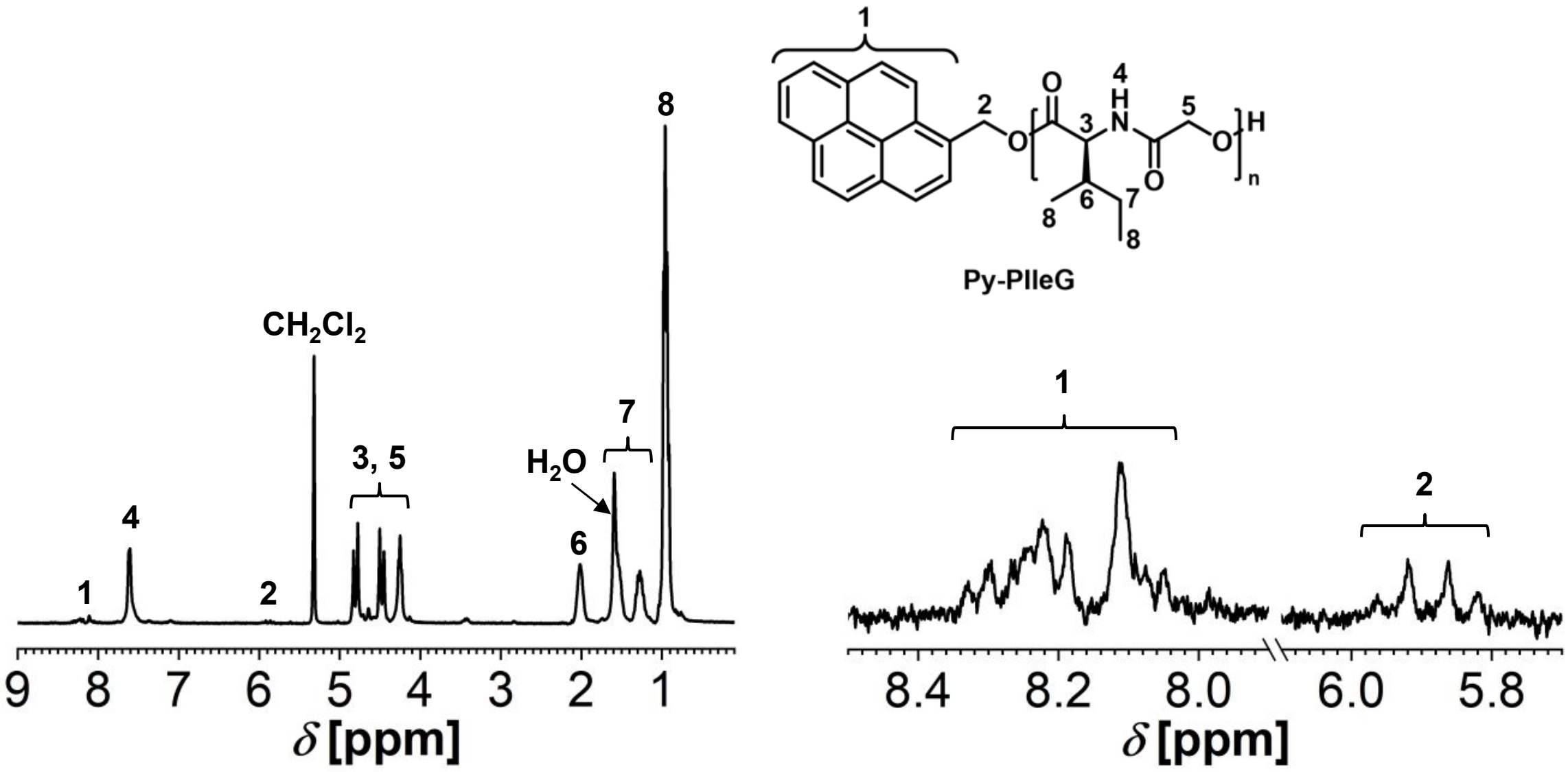

Figure S5. ^{1}H NMR spectrum (300 MHz, CD_2Cl_2) of **Py-PIleG** and zoom into the region of the α-end group signals.

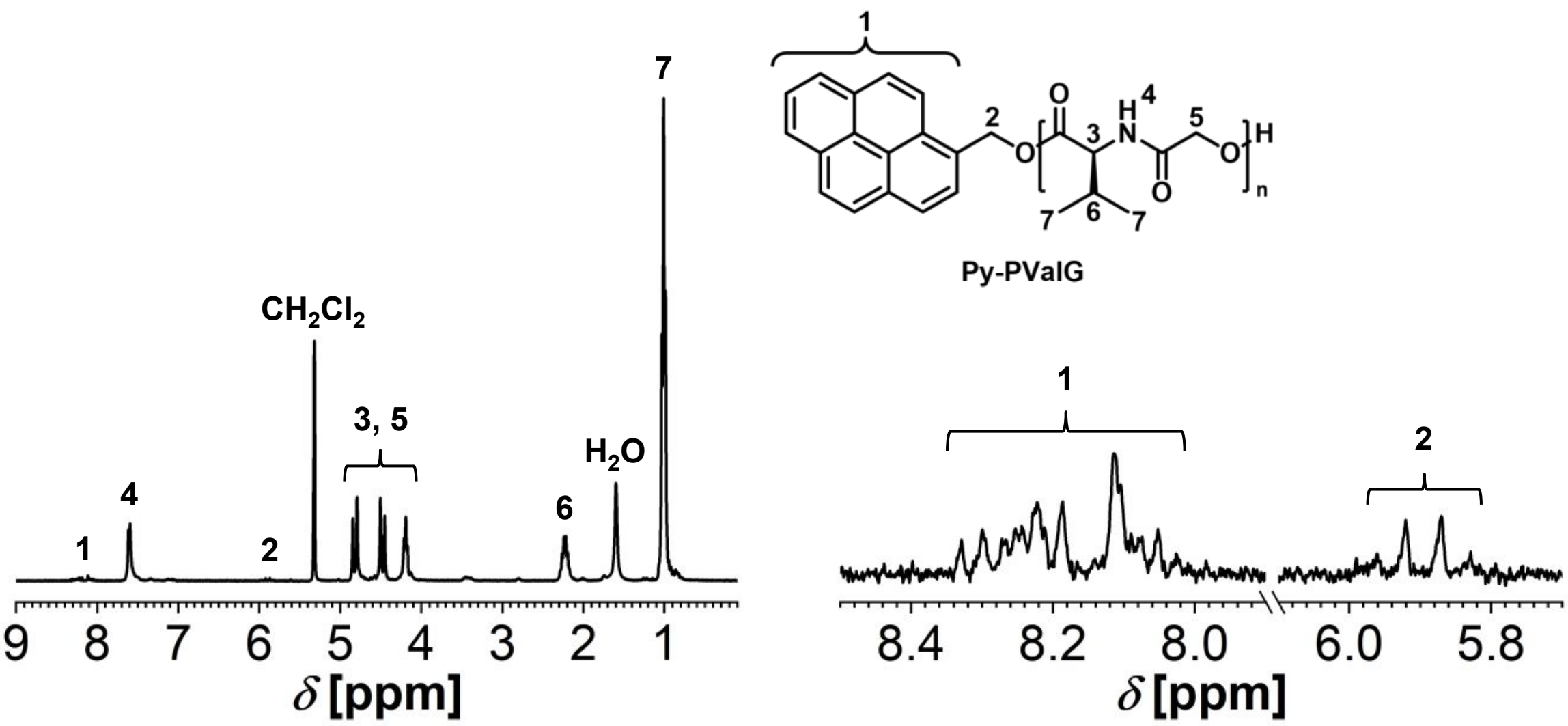

Figure S6. ^{1}H NMR spectrum (300 MHz, CD_2Cl_2) of **Py-PValG** and zoom into the region of the α-end group signals.

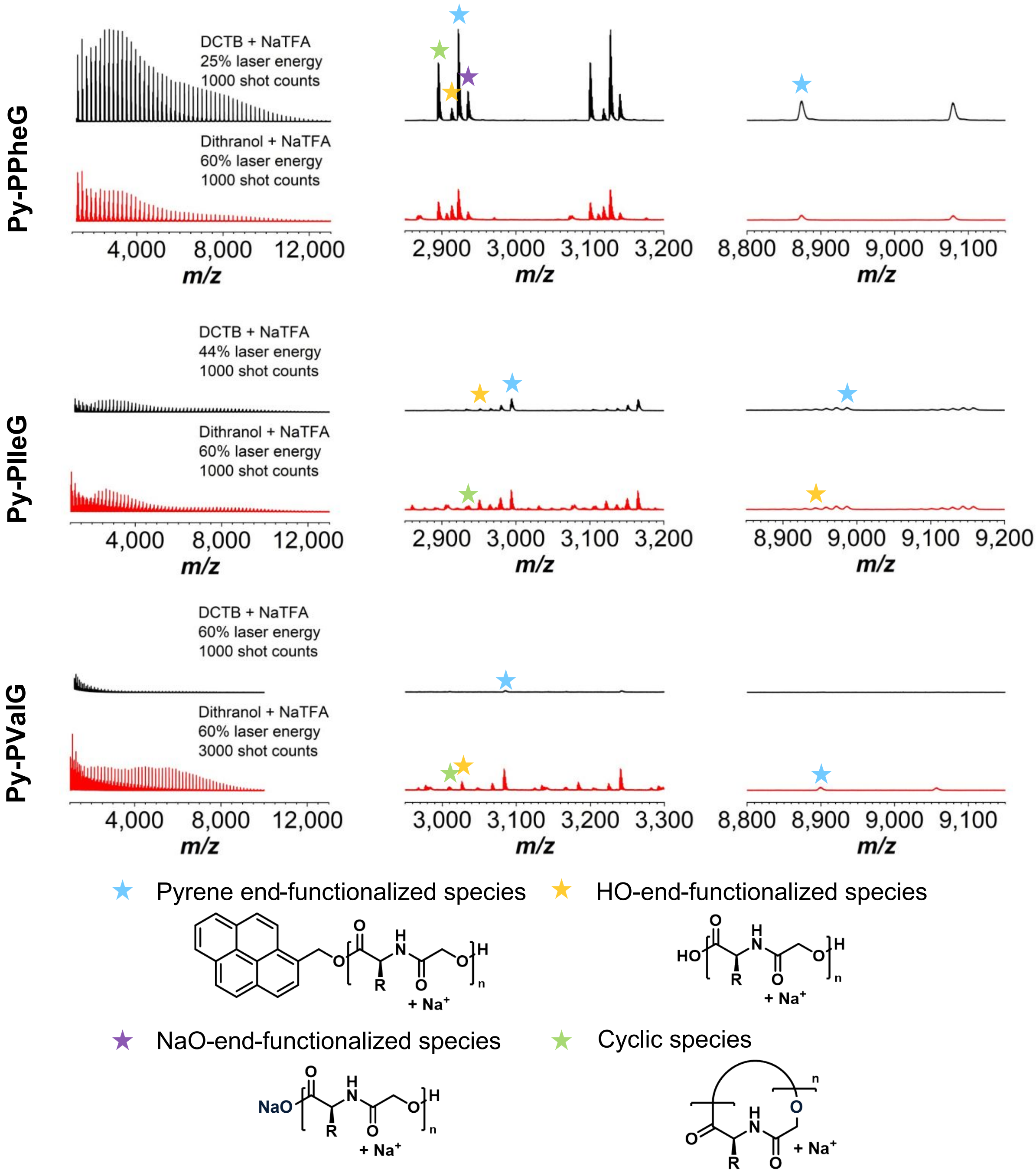

Figure S7. Influence of the matrix on ionization of the Py-PEAs in MALDI-TOF MS. Left: Full mass spectra obtained in DCTB or dithranol at the specified measurement conditions for **Py-PPheG**, **Py-PIleG** and **Py-PValG**. Middle and right: Zoom into the spectra at *m/z* ratios of around 3,000 and 9,000 with constant scaling of the ordinate axis and assignment of the signals to the species indicated below.

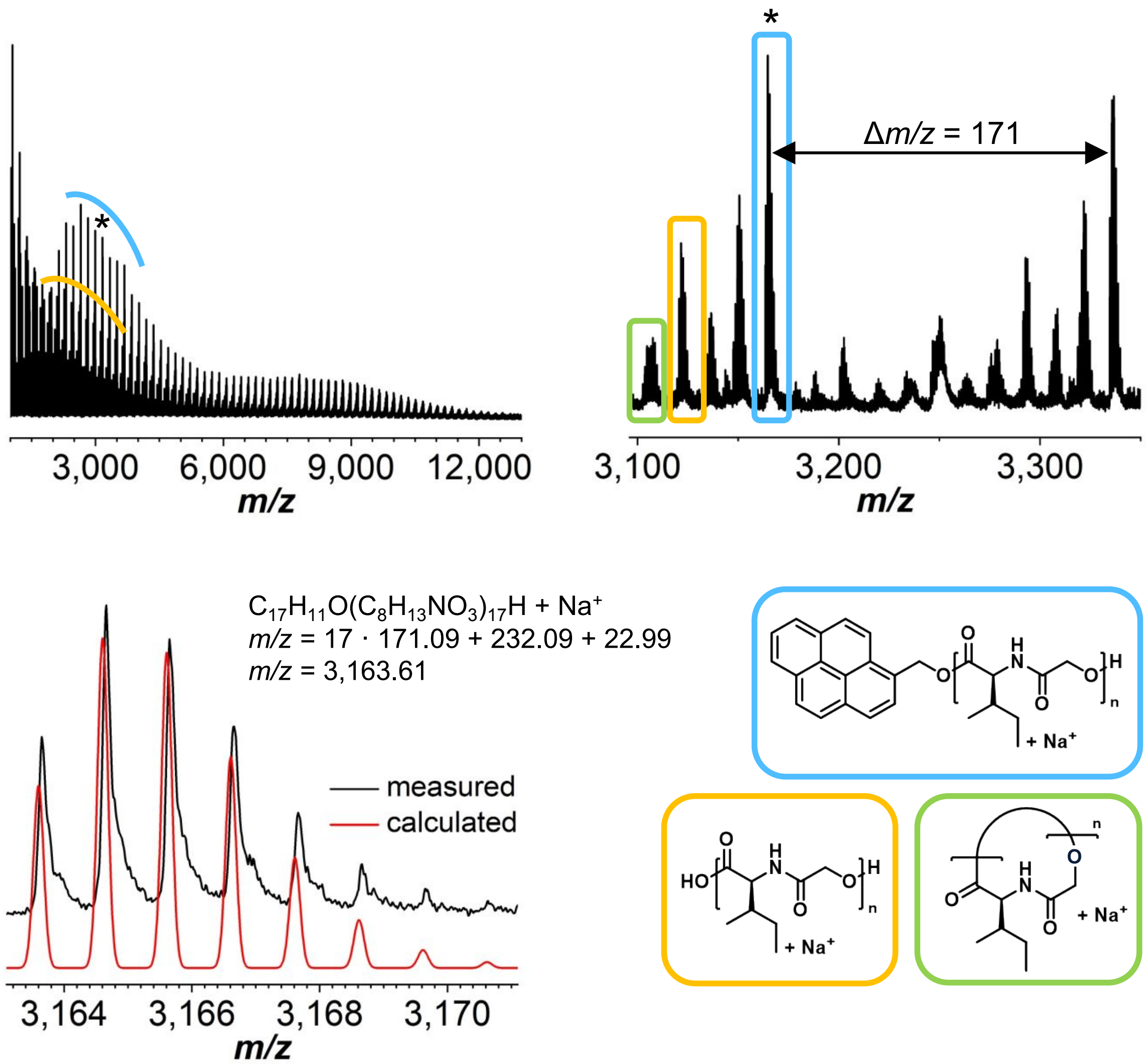

Figure S8. MALDI-TOF MS analysis of **Py-PIleG** (dithranol, NaTFA). Top left: Full mass spectrum of **Py-PIleG** with highlighted distribution of the identified species. Top right: Zoom into the spectrum with marked signals of the identified species. Bottom left: Overlay of the measured and the calculated isotopic pattern of the most abundant identified species. Bottom right: Overview of the identified species.

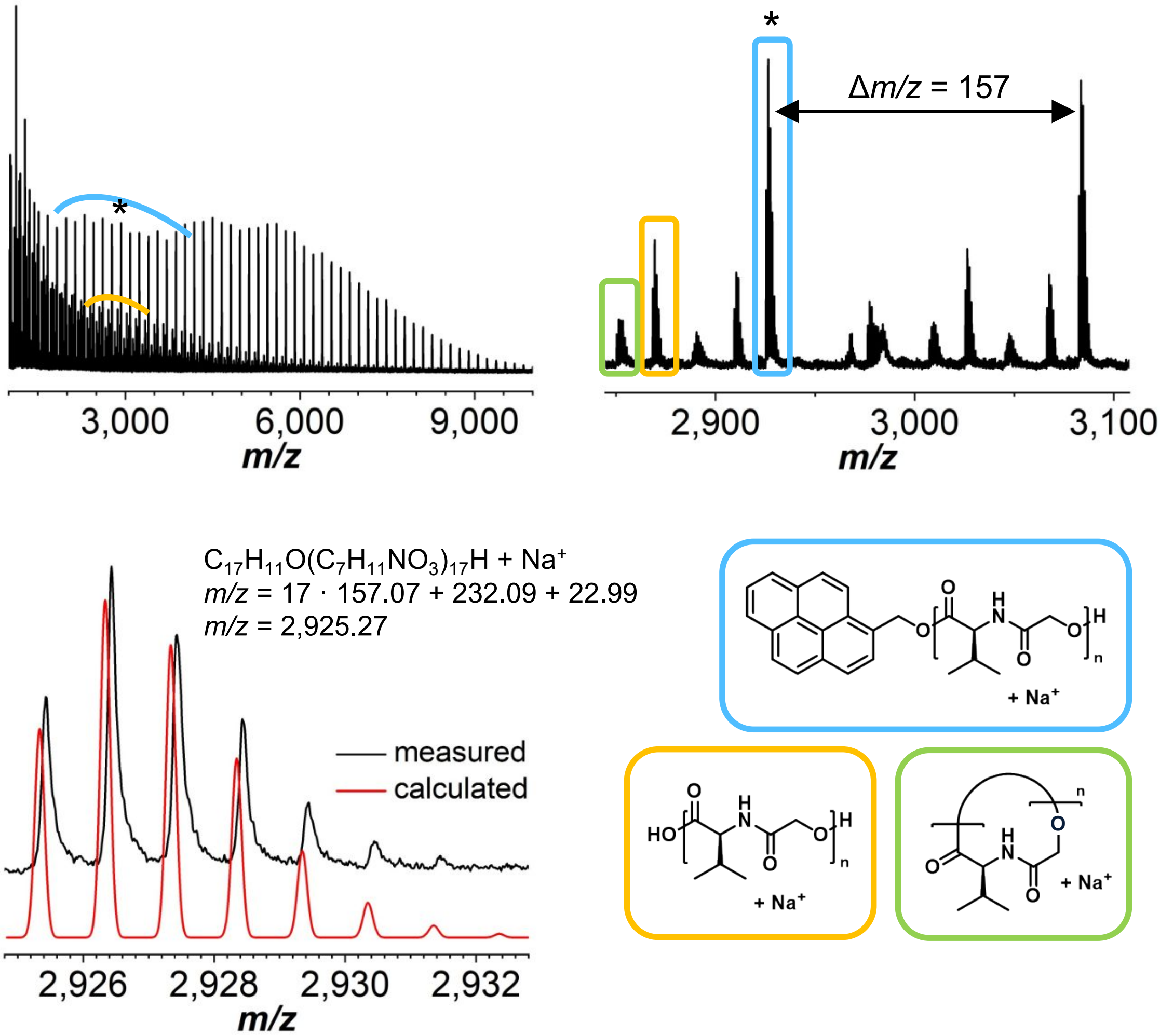

Figure S9. MALDI-TOF MS analysis of **Py-PValG** (dithranol, NaTFA). Top left: Full mass spectrum of **Py-PValG** with highlighted distribution of the identified species. Top right: Zoom into the spectrum with marked signals of the identified species. Bottom left: Overlay of the measured and the calculated isotopic pattern of the most abundant identified species. Bottom right: Overview of the identified species.

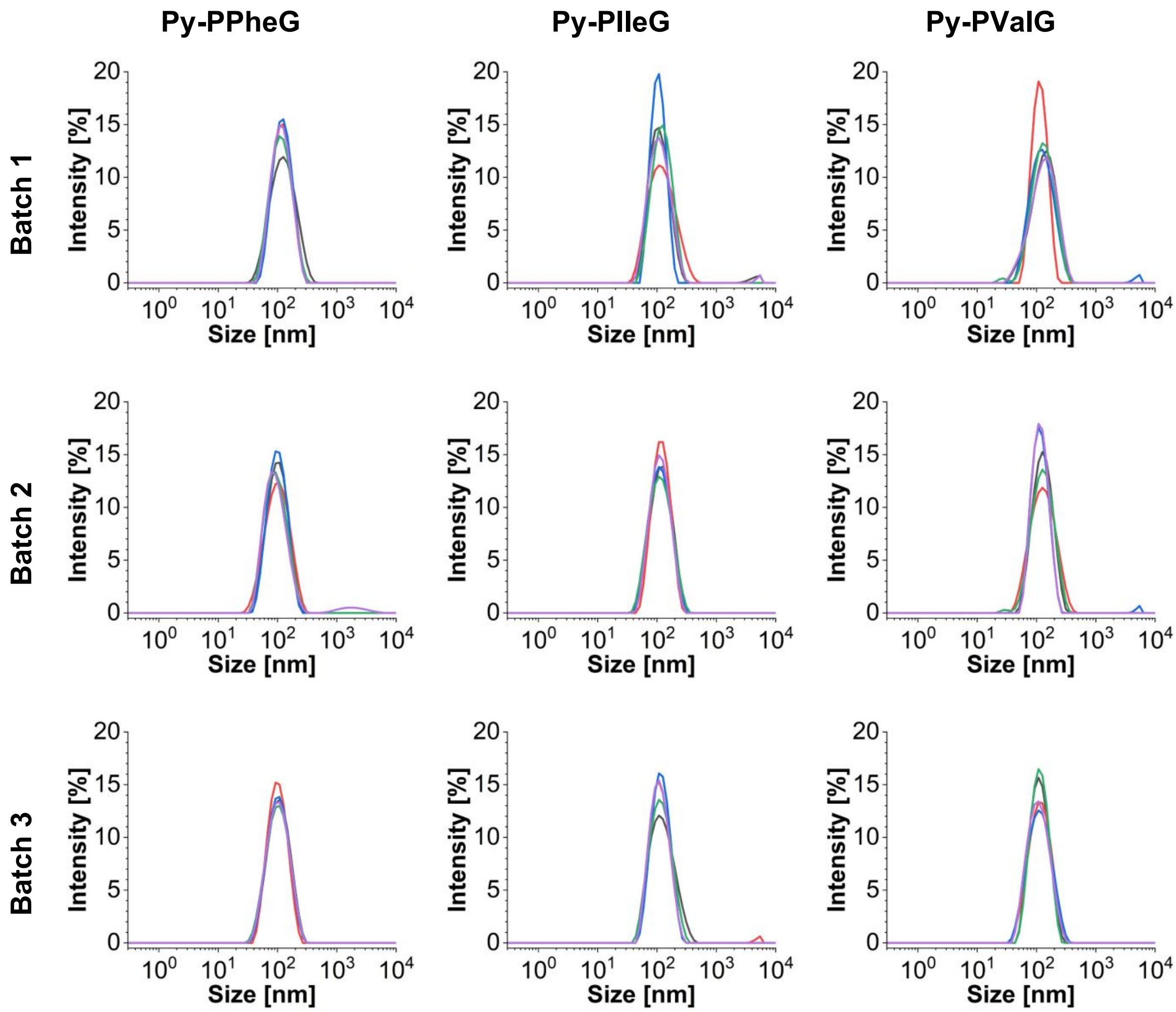

Figure S10. Hydrodynamic diameter distributions plotted against intensity obtained from the DLS measurements of the **Py-PPheG**-, **Py-PIleG**- and **Py-PValG**-based NPs formulated without PVA (n = 3, five runs per measurement).

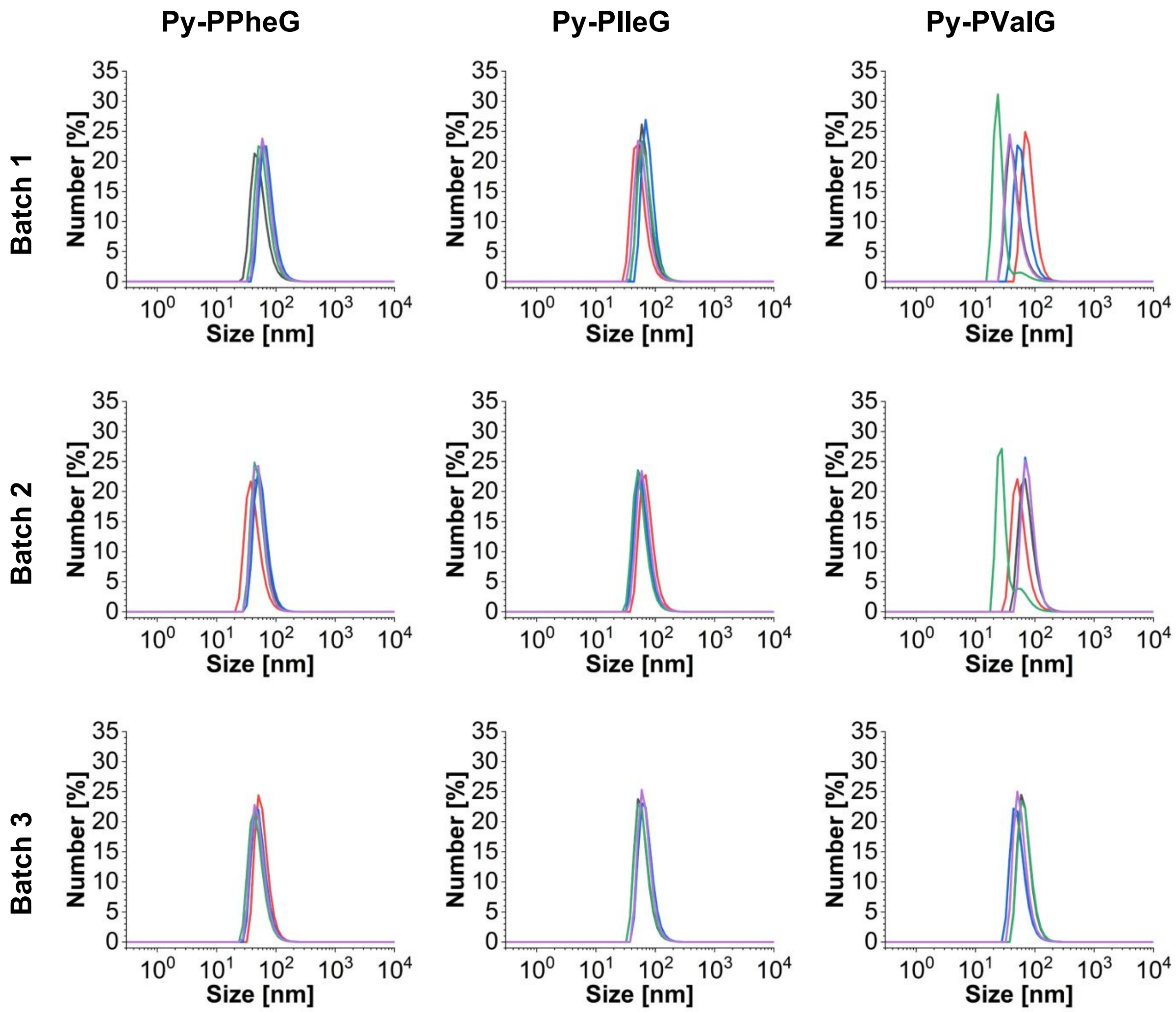

Figure S11. Hydrodynamic diameter distributions plotted against number obtained from the DLS measurements of the **Py-PPheG**-, **Py-PIleG**- and **Py-PValG**-based NPs formulated without PVA (n = 3, five runs per measurement).

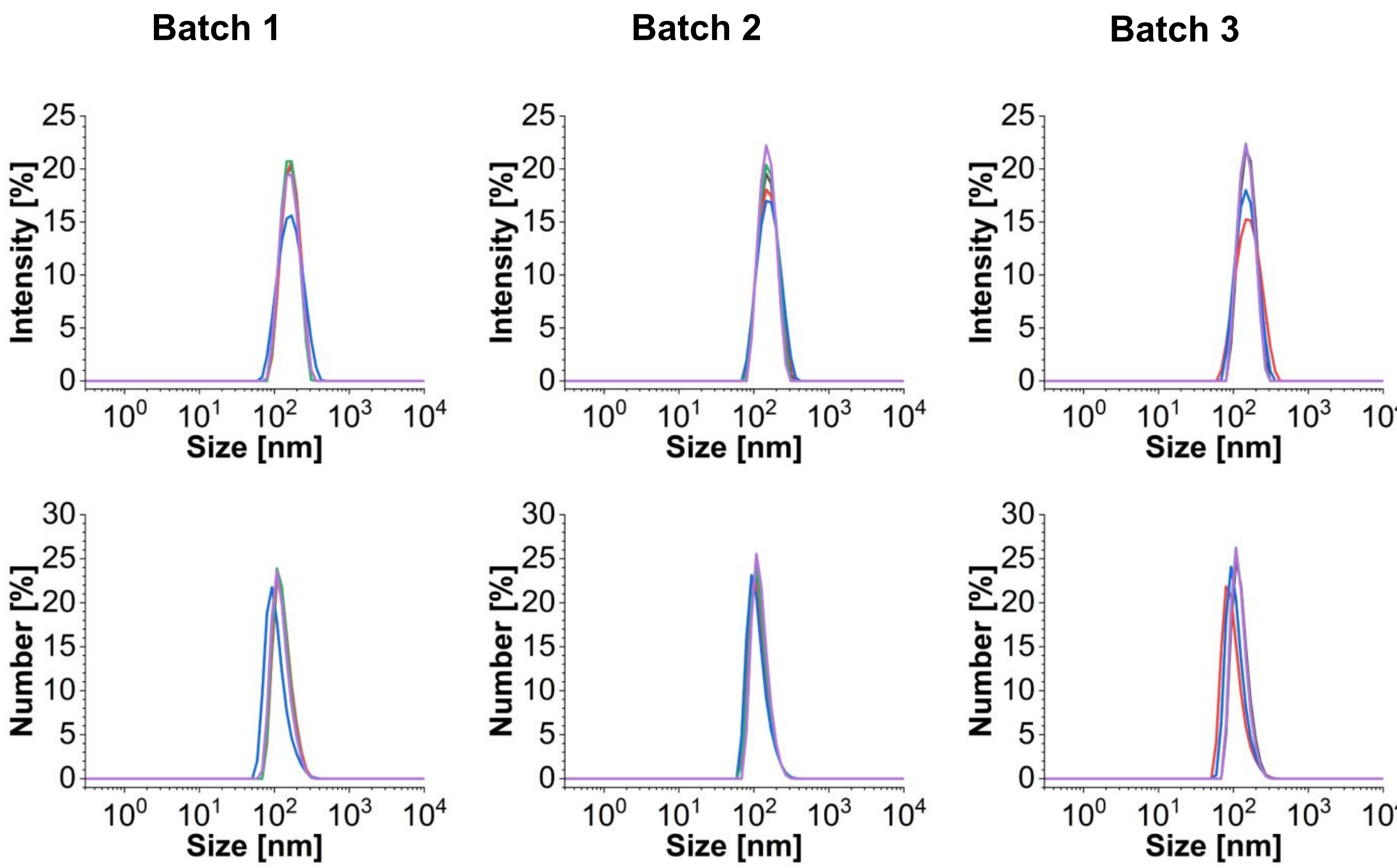

Figure S12. Hydrodynamic diameter distributions plotted against intensity or number obtained from the DLS measurements of the **Py-PPheG**-based NPs formulated with PVA (n = 3, five runs per measurement). The corresponding NPs were used for initial degradation tests using proteinase K or NaOH.

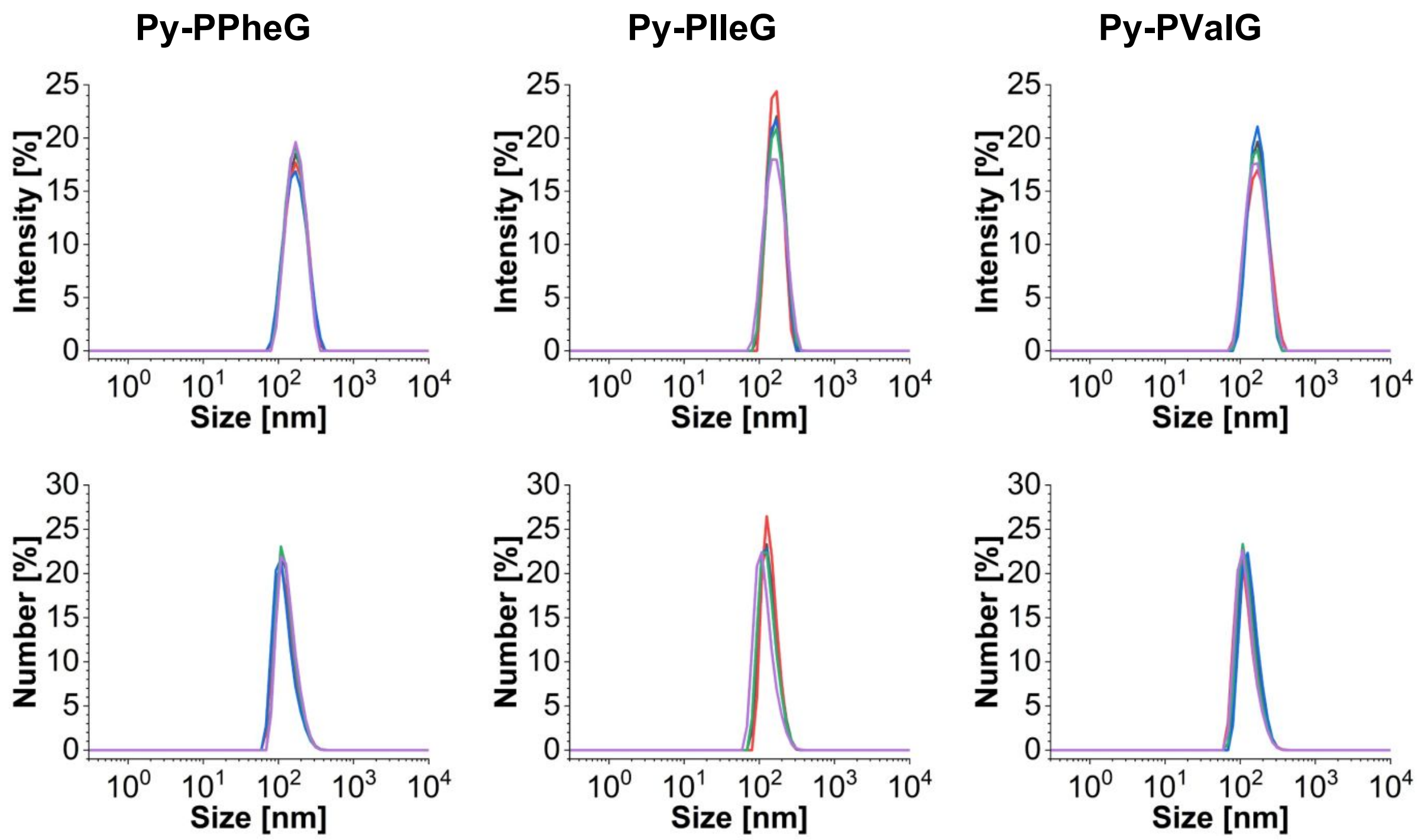

Figure S13. Hydrodynamic diameter distributions plotted against intensity or number obtained from the DLS measurements of the **Py-PPheG-**, **Py-PIleG-** and **Py-PValG**-based NPs formulated with PVA and (n = 1, five runs per measurement). The corresponding NPs were used for degradation kinetic studies using proteinase K.

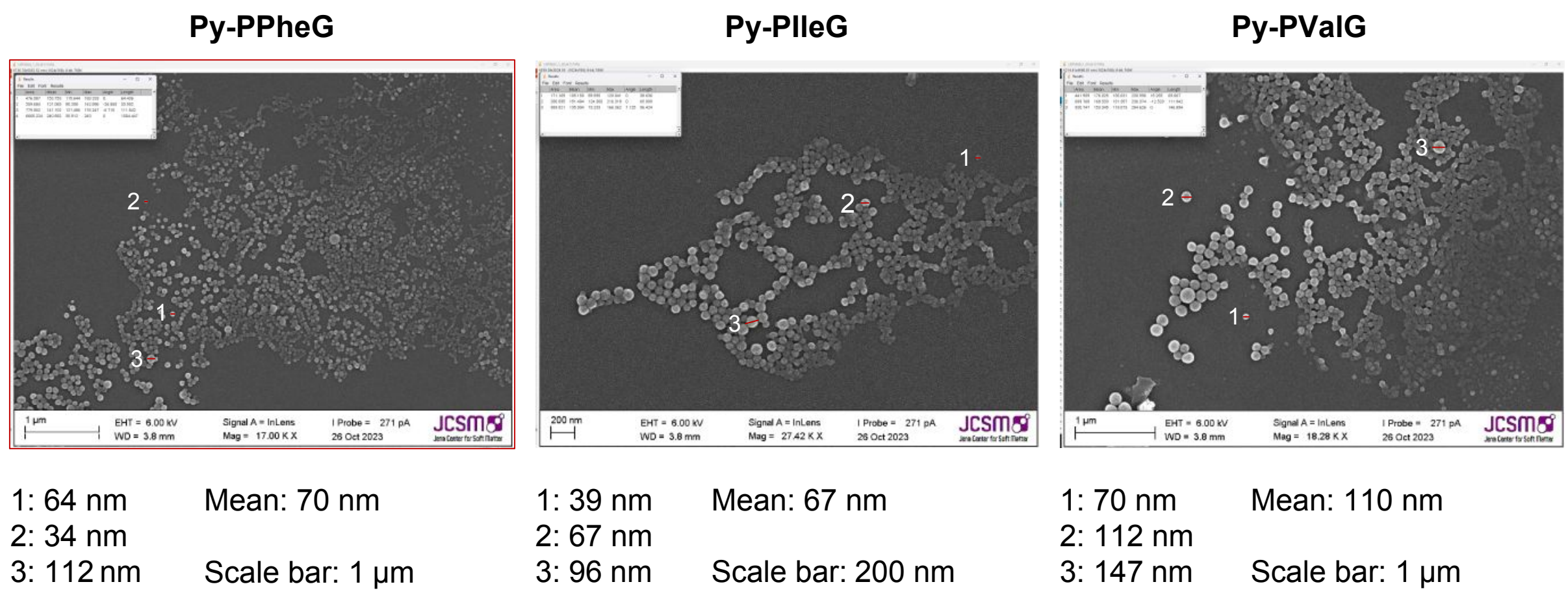

Figure S14. Screenshots of the evaluation of NP diameters from the SEM images using the ImageJ software with highlights of the corresponding NPs.

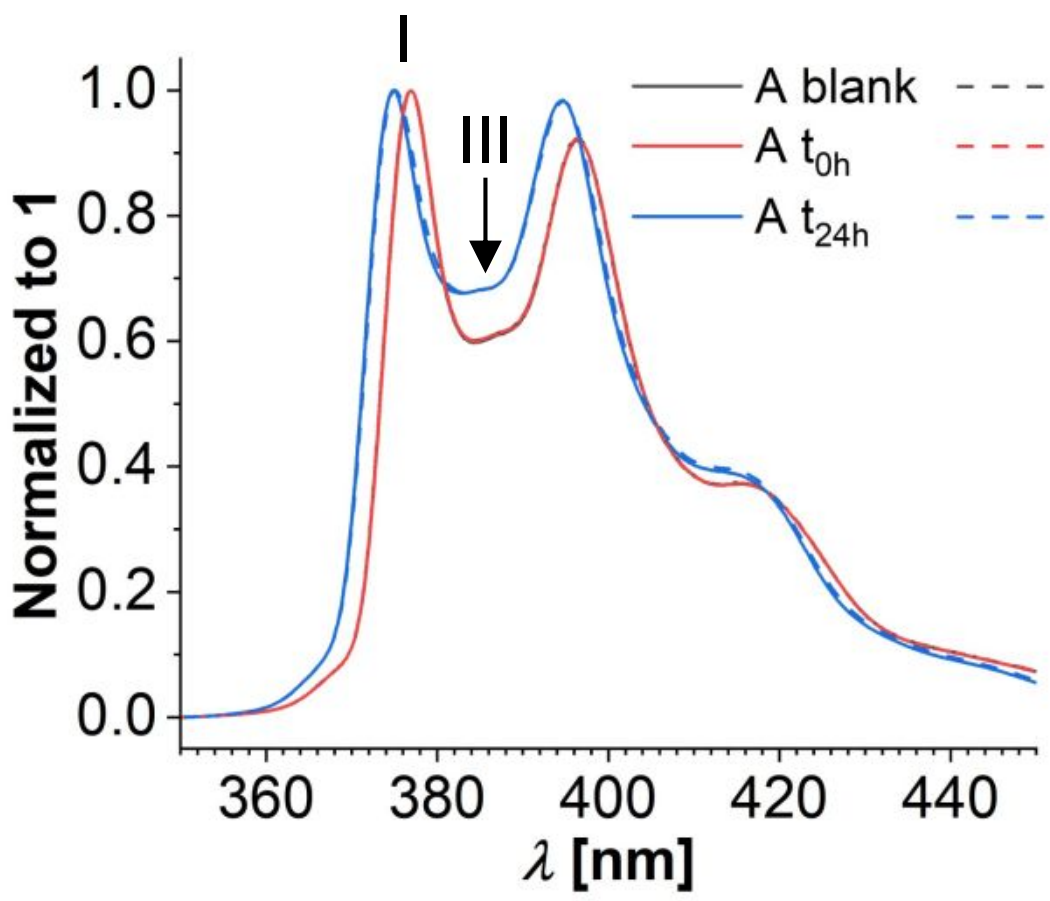

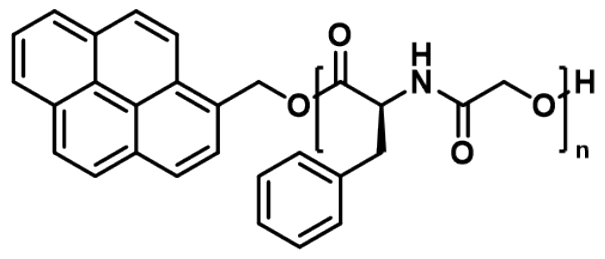

Py-PPheG

	I_{III}/I_I (A)	I_{III}/I_I (B)	I_{III}/I_I (C)
**blank (H_2O)**	0.61	0.61	0.61
**t_{0h} (NaOH)**	0.61	0.61	0.61
**t_{24h} (NaOH)**	0.69	0.69	0.69

Figure S15. NP degradation test in 0.002 M aqueous NaOH solution using three batches of **Py-PPheG** NP (A, B, C) at a NP concentration of 6.8 µg mL^{-1}. Spectra were recorded directly after NaOH addition (t_{0h}) and after 24 hours (t_{24h}) at 25 °C. Spectra of blanks were measured in pure water and overlap with the spectra at t_{0h}. The samples were stored at 37 °C between the measurements. Left: Fluorescence spectra (excitation at 339 nm). Right: Intensity ratios of band III at 387 nm and band I at about 376 nm.

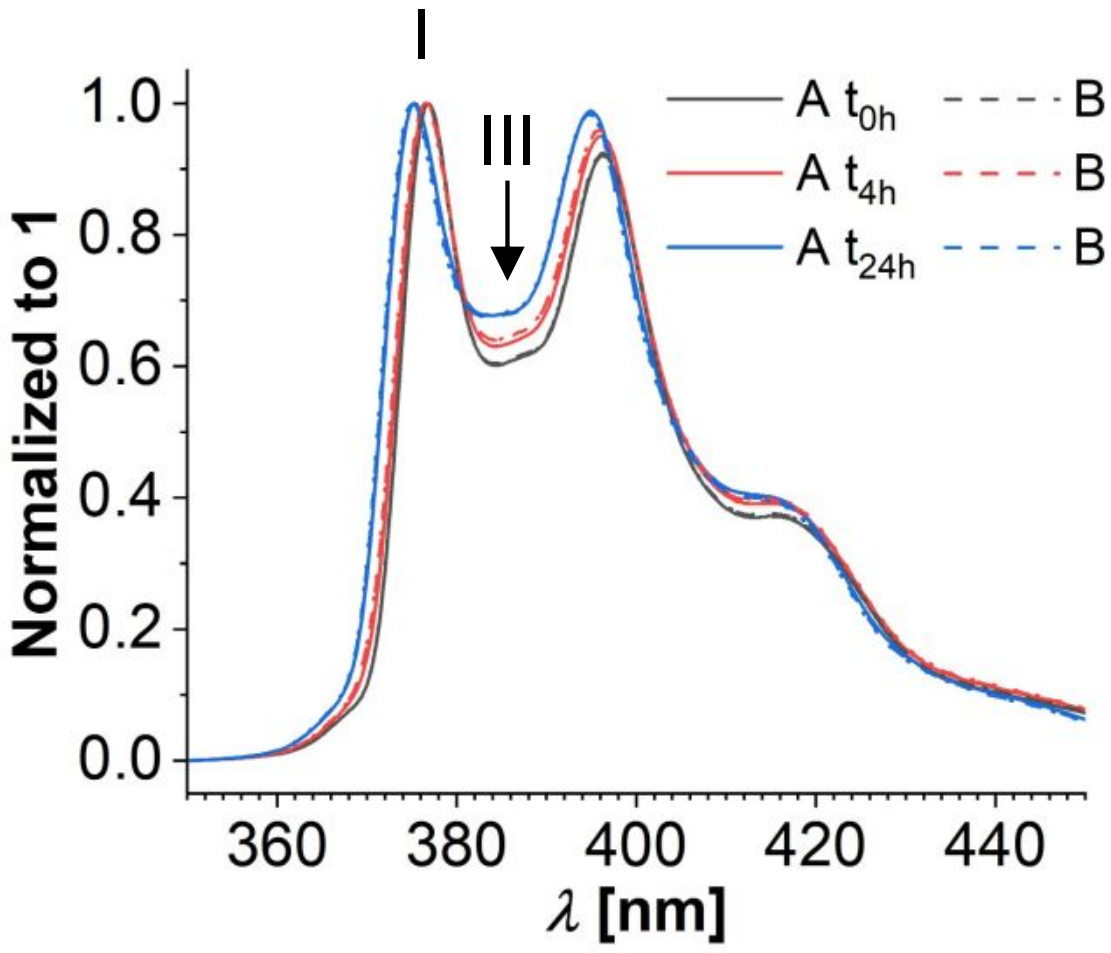

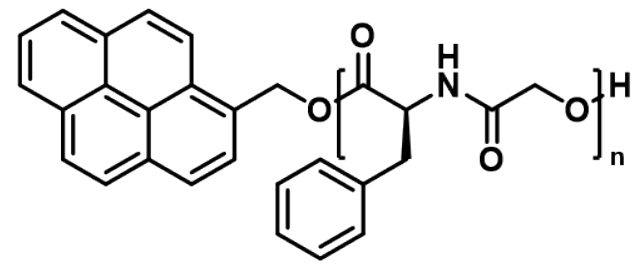

Py-PPheG

	I_{III}/I_{I} (A)	I_{III}/I_{I} (B)	I_{III}/I_{I} (C)
**t_{0h} (PK)**	0.61	0.62	0.62
**t_{4h} (PK)**	0.64	0.65	0.65
**t_{24h} (PK)**	0.69	0.69	0.69

Figure S16. NP degradation test with proteinase K (PK, c_{PK} = 167 µg mL^{-1}) using three batches of **Py-PPheG** NP (A, B, C) at a NP concentration of 6.8 µg mL^{-1} in purified water. Spectra were recorded directly after PK addition (t_{0h}), after 4 hours (t_{4h}) and after 24 hours (t_{24h}) at 25 °C. The samples were stored at 37 °C between the measurements. Left: Fluorescence spectra (excitation at 339 nm). Right: Intensity ratios of band III at 387 nm and band 1 at about 376 nm.

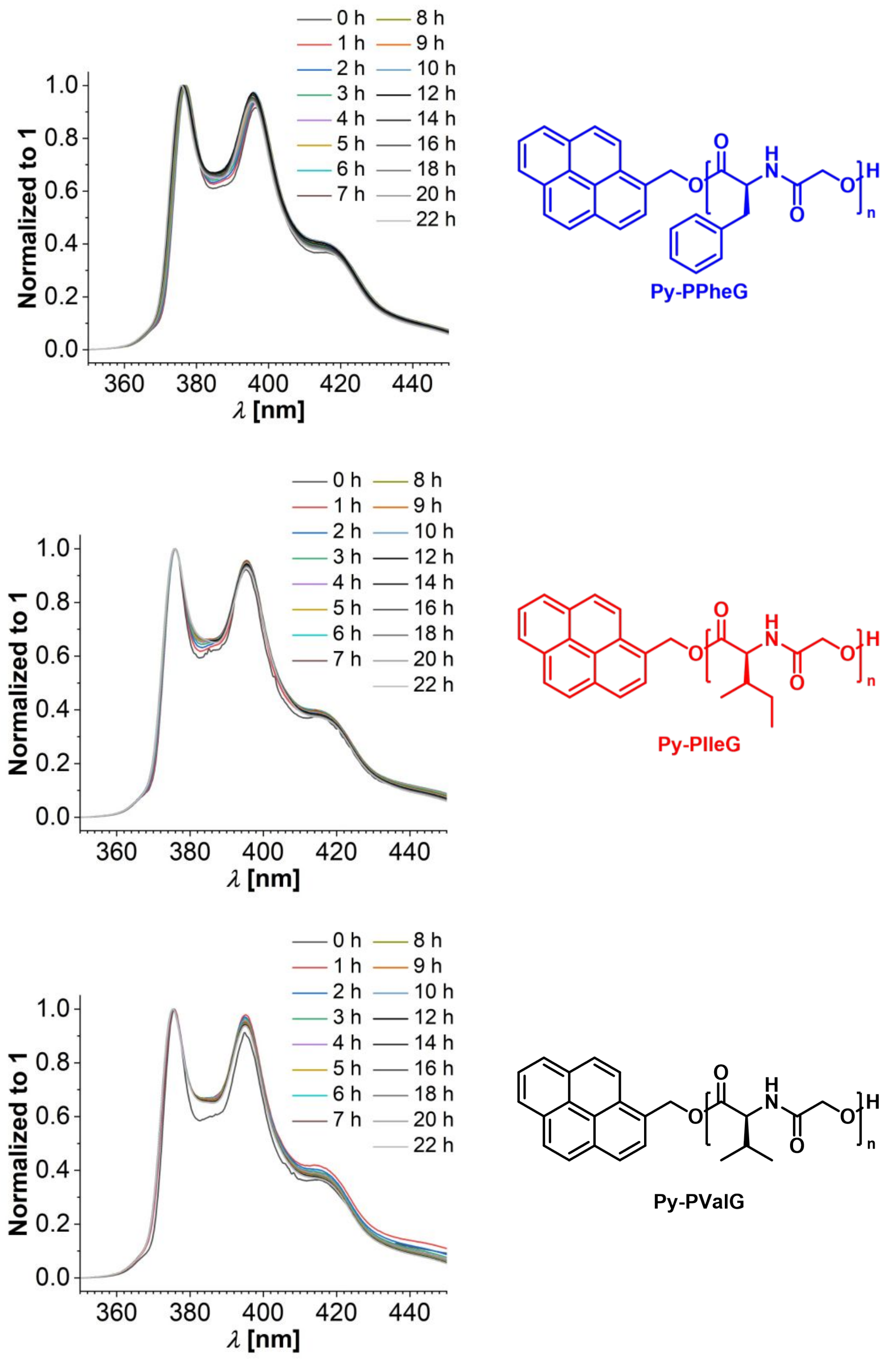

Figure S17. Overlay of the fluorescence spectra obtained from the degradation kinetic studies of the NPs based on **Py-PPheG**, **Py-PIleG** and **Py-PValG** using proteinase K. The measurements were performed in purified water at 37 ˚C (c_{NP} = 6.5 μg mL^{-1}, c_{PK} = 167 μg mL^{-1}, excitation at 339 nm).

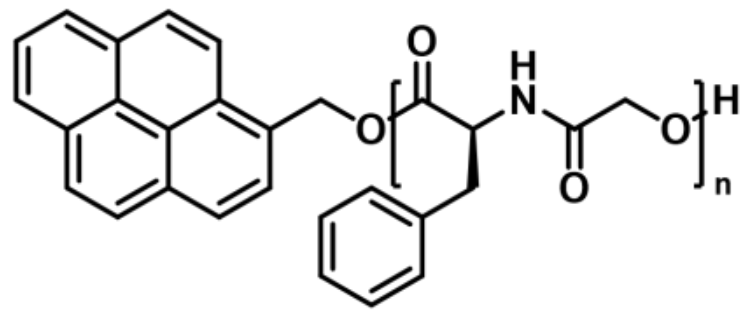

Py-PPheG

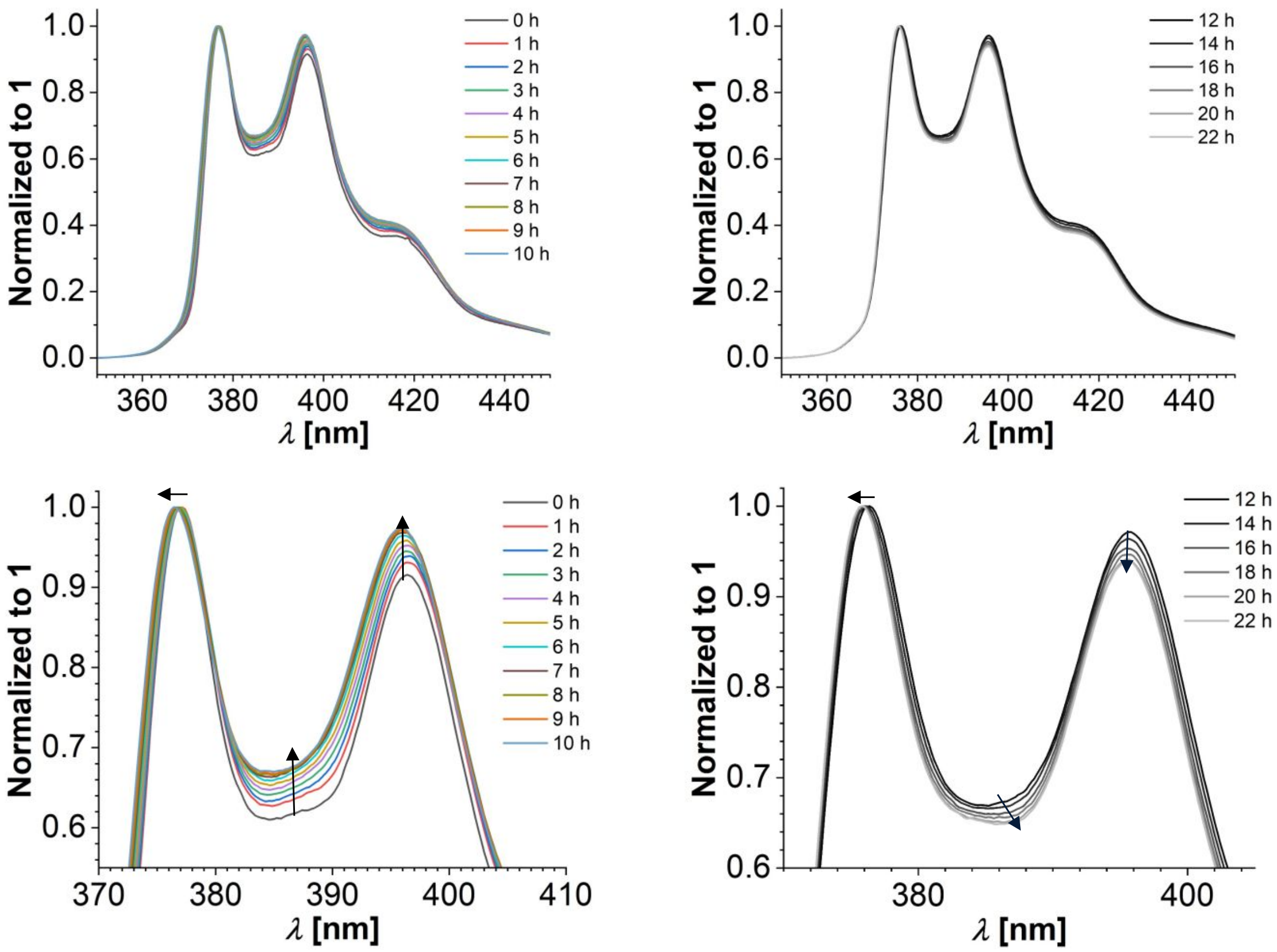

Figure S18. Overview of the fluorescence spectra received from the degradation study of **Py-PPheG** NPs using proteinase K in purified water (c_{NP} = 6.5 µg mL^{-1}, c_{PK} = 167 µg mL^{-1}, excitation at 339 nm, 37 °C). Top left: Overlay of the spectra of every hour from the beginning to 10 h. Bottom left: Zoom into the spectra up to 10 h and highlighted spectral changes over time. Top right: Overlay of the spectra of every second hour from 12 to 22 h measurement time. Bottom right: Zoom into the spectra from 12 to 22 h and highlighted spectral changes over time.

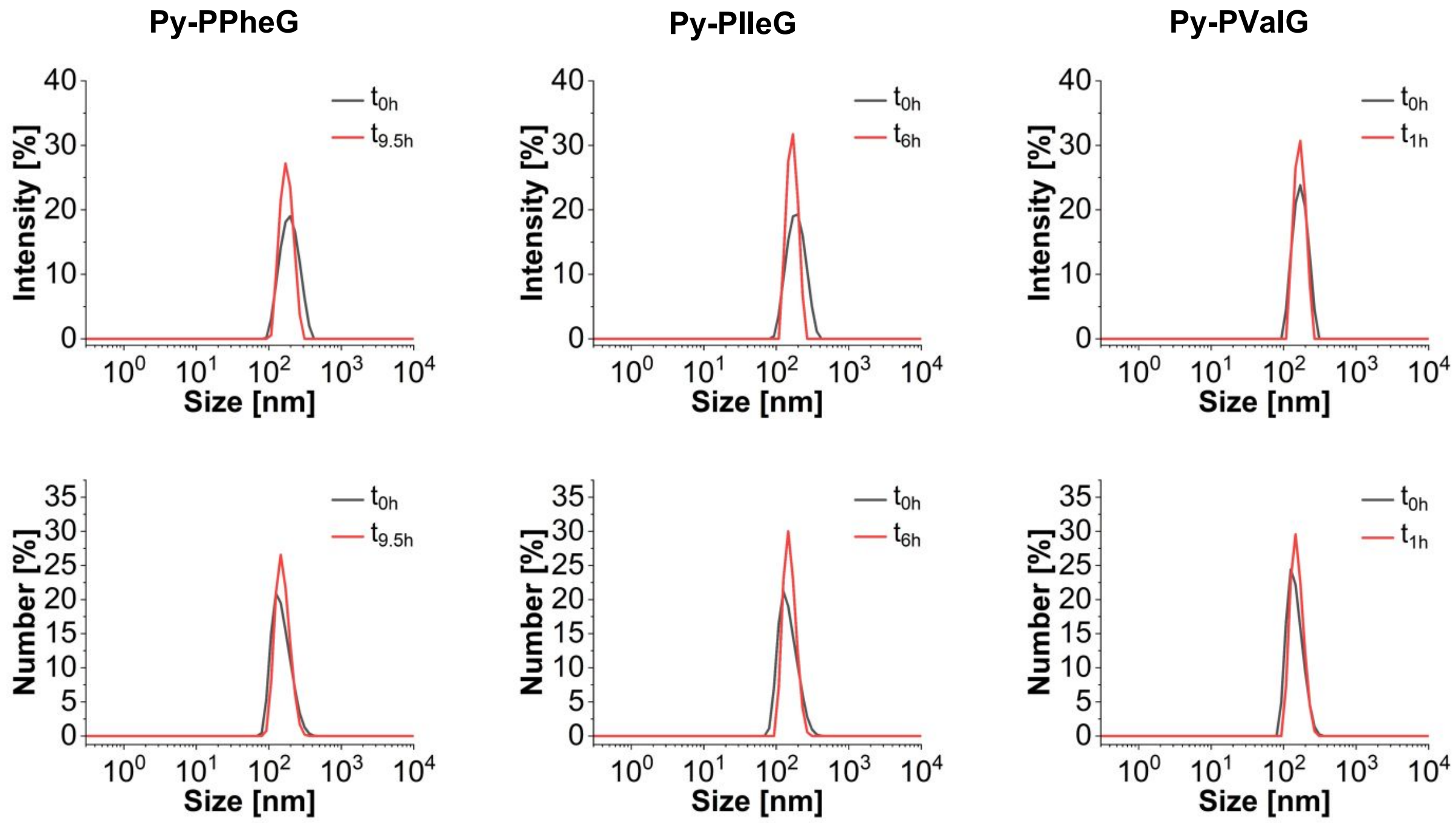

Figure S19. Selected hydrodynamic diameter distributions plotted against intensity or number obtained from the DLS measurements during the degradation kinetic studies of the **Py-PPheG-**, **Py-PIleG**- and **Py-PValG**-based NPs (one run per measurement).

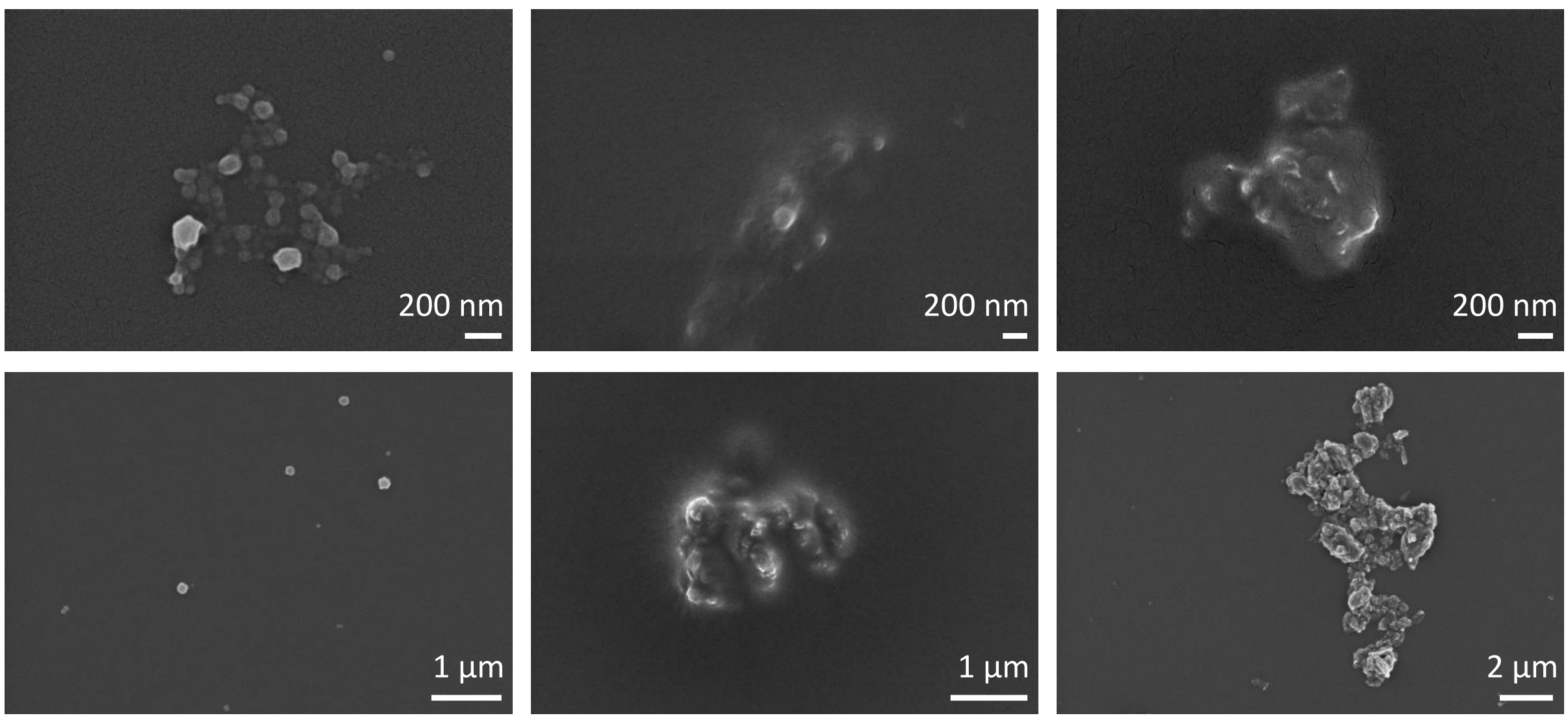

Figure S20. Representative SEM images of degraded **Py-PValG** NPs 1 h after treatment with proteinase K at 37 °C.

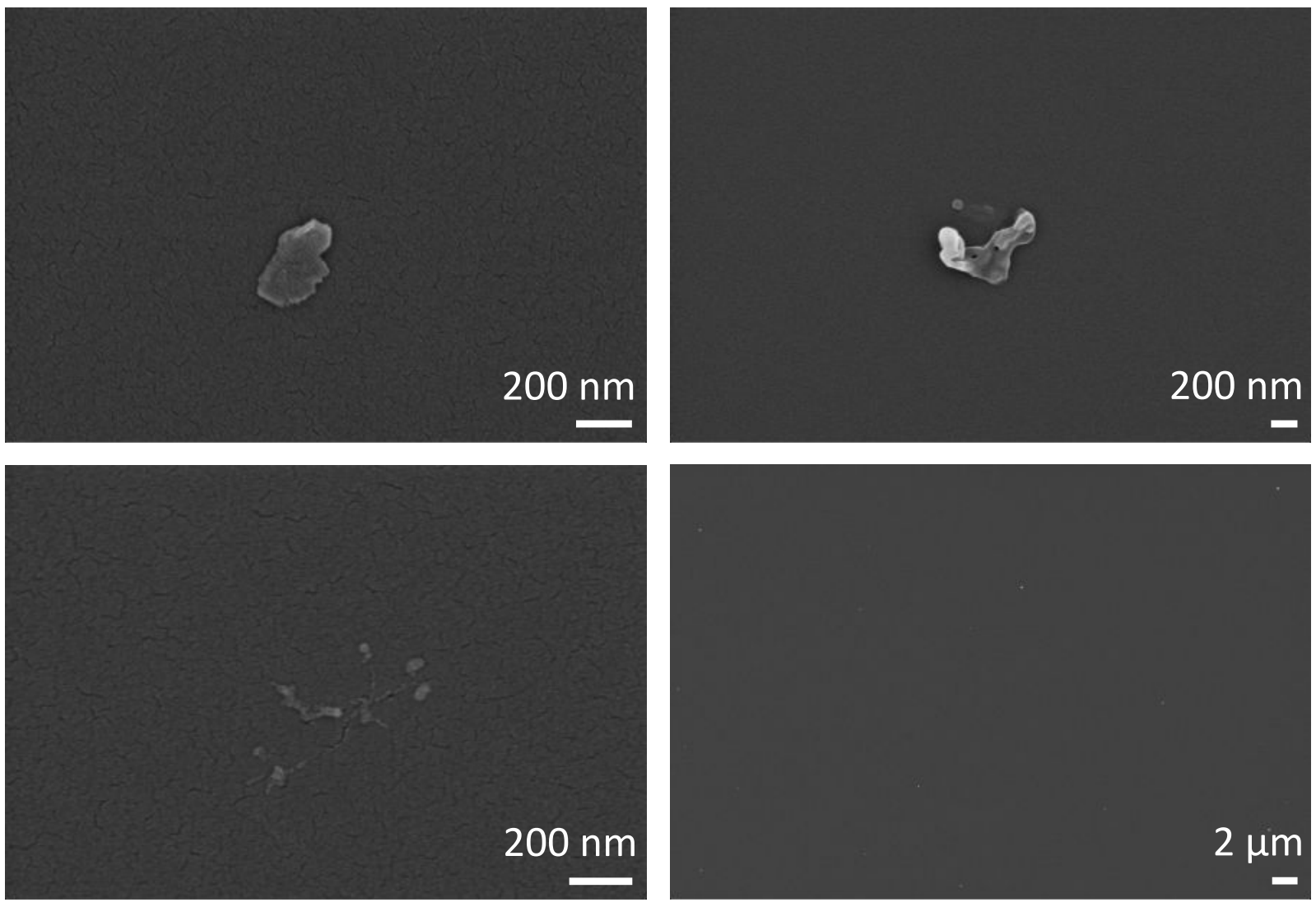

Figure S21. Representative SEM images of degraded **Py-PValG** NPs 3 h after treatment with proteinase K at 37 °C.

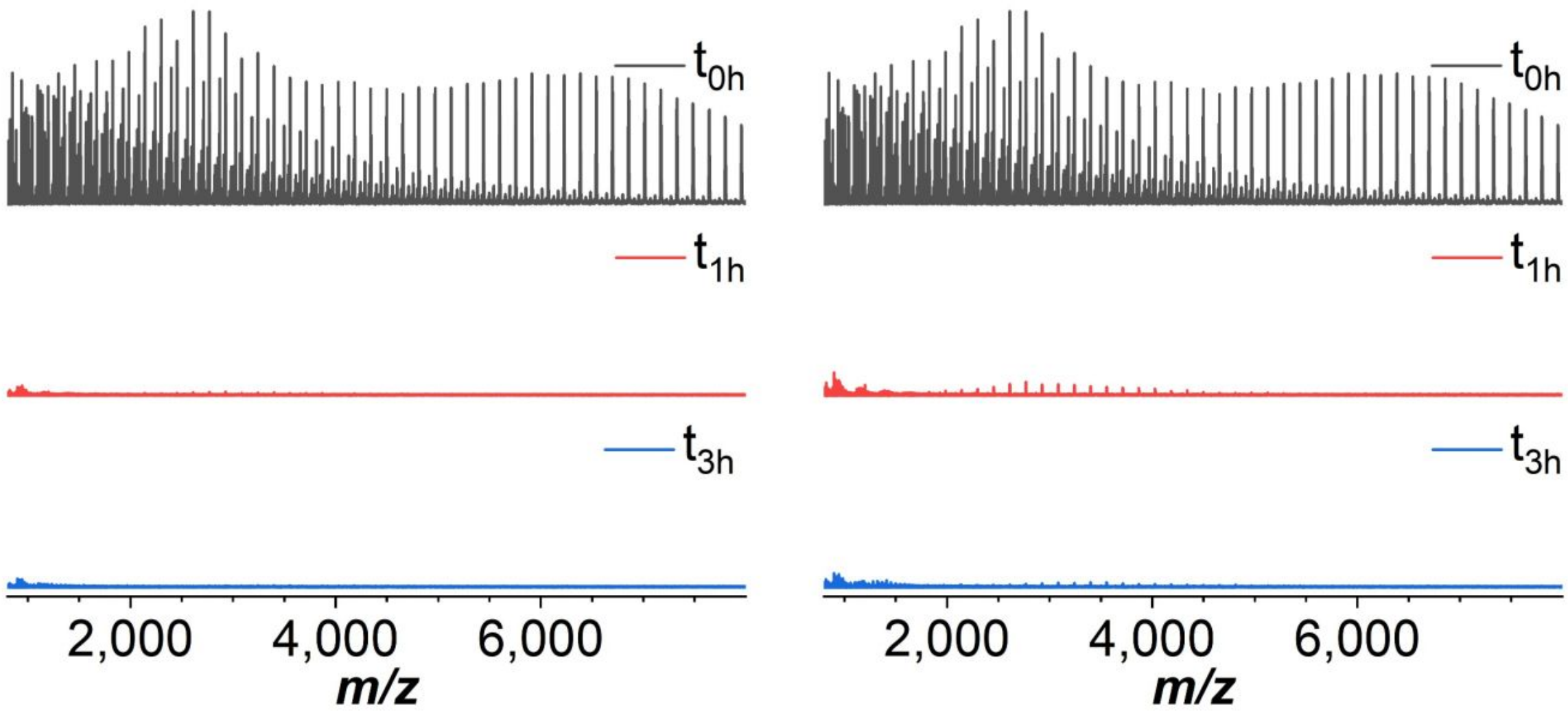

Figure S22. MALDI-TOF mass spectra (dithranol, NaTFA, 60% laser energy, 1,000 shots) of degraded **Py-PValG** NPs. Lyophilized samples were measured 1 h or 3 h after treatment with proteinase K at 37 °C. The spectra on the left- and right-hand side were measured from the same sample at different laser positions.

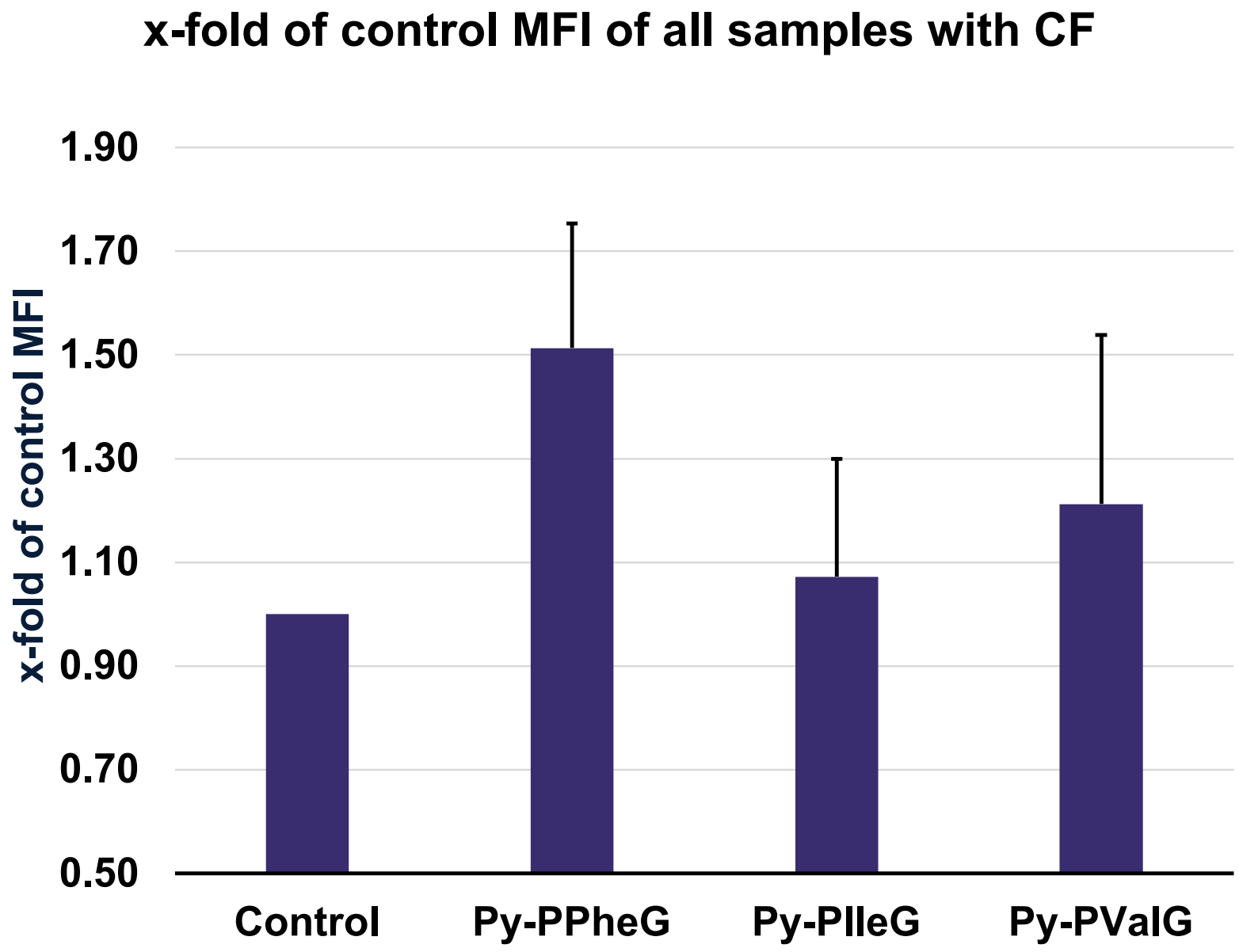

Figure S23. Mean fluorescence intensity (MFI) of L929 cells treated with Py-PEA NP suspension expressed as x-fold changes compared to untreated control cells. The fluorescence intensity of the individual NP suspensions at 415 nm was used as a correction factor (CF).
